# Multidrug Resistant Pulmonary Tuberculosis Treatment Regimens and Patient Outcomes: An Individual Patient Data Meta-analysis of 9,153 Patients

**DOI:** 10.1371/journal.pmed.1001300

**Published:** 2012-08-28

**Authors:** Shama D. Ahuja, David Ashkin, Monika Avendano, Rita Banerjee, Melissa Bauer, Jamie N. Bayona, Mercedes C. Becerra, Andrea Benedetti, Marcos Burgos, Rosella Centis, Eward D. Chan, Chen-Yuan Chiang, Helen Cox, Lia D'Ambrosio, Kathy DeRiemer, Nguyen Huy Dung, Donald Enarson, Dennis Falzon, Katherine Flanagan, Jennifer Flood, Maria L. Garcia-Garcia, Neel Gandhi, Reuben M. Granich, Maria G. Hollm-Delgado, Timothy H. Holtz, Michael D. Iseman, Leah G. Jarlsberg, Salmaan Keshavjee, Hye-Ryoun Kim, Won-Jung Koh, Joey Lancaster, Christophe Lange, Wiel C. M. de Lange, Vaira Leimane, Chi Chiu Leung, Jiehui Li, Dick Menzies, Giovanni B. Migliori, Sergey P. Mishustin, Carole D. Mitnick, Masa Narita, Philly O'Riordan, Madhukar Pai, Domingo Palmero, Seung-kyu Park, Geoffrey Pasvol, Jose Peña, Carlos Pérez-Guzmán, Maria I. D. Quelapio, Alfredo Ponce-de-Leon, Vija Riekstina, Jerome Robert, Sarah Royce, H. Simon Schaaf, Kwonjune J. Seung, Lena Shah, Tae Sun Shim, Sonya S. Shin, Yuji Shiraishi, José Sifuentes-Osornio, Giovanni Sotgiu, Matthew J. Strand, Payam Tabarsi, Thelma E. Tupasi, Robert van Altena, Martie Van der Walt, Tjip S. Van der Werf, Mario H. Vargas, Pirett Viiklepp, Janice Westenhouse, Wing Wai Yew, Jae-Joon Yim, Collaborative Group for Meta-Analysis of Individual Patient Data in MDR-TB, Shama D. Ahuja, David Ashkin, Monika Avendano, Rita Banerjee, Melissa Bauer, Jamie N. Bayona, Mercedes C. Becerra, Andrea Benedetti, Marcos Burgos, Rosella Centis, Eward D. Chan, Chen-Yuan Chiang, Helen Cox, Lia D'Ambrosio, Kathy DeRiemer, Nguyen Huy Dung, Donald Enarson, Dennis Falzon, Katherine Flanagan, Jennifer Flood, Maria L. Garcia-Garcia, Neel Gandhi, Reuben M. Granich, Maria G. Hollm-Delgado, Timothy H. Holtz, Michael D. Iseman, Leah G. Jarlsberg, Salmaan Keshavjee, Hye-Ryoun Kim, Won-Jung Koh, Joey Lancaster, Christophe Lange, Wiel C. M. de Lange, Vaira Leimane, Chi Chiu Leung, Jiehui Li, Dick Menzies, Giovanni B. Migliori, Sergey P. Mishustin, Carole D. Mitnick, Masa Narita, Philly O'Riordan, Madhukar Pai, Domingo Palmero, Seung-kyu Park, Geoffrey Pasvol, Jose Peña, Carlos Pérez-Guzmán, Maria I. D. Quelapio, Alfredo Ponce-de-Leon, Vija Riekstina, Jerome Robert, Sarah Royce, H. Simon Schaaf, Kwonjune J. Seung, Lena Shah, Tae Sun Shim, Sonya S. Shin, Yuji Shiraishi, José Sifuentes-Osornio, Giovanni Sotgiu, Matthew J. Strand, Payam Tabarsi, Thelma E. Tupasi, Robert van Altena, Martie Van der Walt, Tjip S. Van der Werf, Mario H. Vargas, Pirett Viiklepp, Janice Westenhouse, Wing Wai Yew

**Affiliations:** Bureau of Tuberculosis, New York, New York, United States of America; A.G. Holley Hospital, Lantana, Florida, United States of America; University of Toronto, Toronto, Canada,; Mayo Clinic, Rochester, Minnesota, United States of America; Montreal Chest Institute, McGill University, Montreal, Canada; The Dartmouth Center for Health Care Delivery Science, Hanover, New Hampshire, United States of America; Harvard Medical School, Boston, Massachusetts, United States of America; Partners in Health, Boston, Massachusetts, United States of America; Montreal Chest Institute, McGill University, Montreal, Canada; University of New Mexico School of Medicine, Albuquerque, New Mexico, United States of America; WHO Collaborating Centre for TB and Lung Diseases, Care and Research Institute, Fondazione S. Maugeri, Tradate, Italy; Denver Veterans Affair Medical Center, Denver, Colorado, United States of America; Wan Fang Hospital, School of Medicine-Taipei Medical University, Taiwan; Médecins Sans Frontières, Capetown, South Africa; WHO Collaborating Centre for TB and Lung Diseases, Care and Research Institute, Fondazione S. Maugeri, Tradate, Italy; UC Davis School of Medicine, Davis, California, United States of America; National TB Control Program, Hanoi, Vietnam; International Union against Tuberculosis and Lung Disease, Paris, France; World Health Organization, Geneva, Switzerland; MRC Laboratories, Banjul, The Gambia; California Department of Public Health, Sacramento, California, United States of America; Instituto Nacional de Salud Pública, Mexico, Mexico; Albert Einstein College of Medicine, Bronx, New York, United States of America; World Health Organization, Geneva, Switzerland; Montreal Chest Institute, McGill University, Montreal, Canada; Thailand MOPH & US CDC Collaboration, Bangkok, Thailand; National Jewish Health, Denver, Colorado, United States of America; University of California, San Francisco, San Francisco, United States of America; Harvard Medical School, Boston, Massachusetts, United States of America; Korea Cancer Center Hospital, Seoul, Korea; Samsung Medical Center, Seoul, Korea; South African Medical Research Council, Pretoria, South Africa; Medical Clinic, Tuberculosis Center Borstel, Borstel, Germany; University Medical Center Groningen, Groningen, The Netherlands; Clinic of Tuberculosis and Lung Diseases, Riga, Latvia; Tuberculosis and Chest Services, Hong Kong; New York City Health and Mental Hygiene, New York, New York, United States of America; Montreal Chest Institute, McGill University, Montreal, Canada; WHO Collaborating Centre for TB and Lung Diseases, Care and Research Institute, Fondazione S. Maugeri, Tradate, Italy; Tomsk Oblast Tuberculosis Dispensary, Tomsk, Russia; Harvard Medical School, Boston, Massachusetts, United States of America; University of Washington, Seattle, Washington, United States of America; City Road Medical Centre, London, United Kingdom; Montreal Chest Institute, McGill University, Montreal, Canada; Hospital F.J. Muñiz, Buenos Aires, Argentina; TB Center, Seoul, Korea; Imperial College London, London, United Kingdom; Universidad Autonoma Madrid, Madrid, Spain; Instituto de Salud del Estado de Aguascalientes, Mexico, Mexico; Tropical Disease Foundation, Makati City, Philippines; Instituto Nacional de Ciencias Médicas y de Nutrición “Salvador Zubirán”, Mexico, Mexico; Clinic of Tuberculosis and Lung Diseases, Riga, Latvia; Bactériologie-Hygiène – UPMC, Paris, France; University of California, San Francisco, San Francisco, United States of America; Stellenbosch University, Stellenbosch, South Africa; Brigham and Women's Hospital, Boston, Massachusetts, United States of America; Montreal Chest Institute, McGill University, Montreal, Canada; University of Ulsan College of Medicine, Seoul, Korea; Brigham and Women's Hospital, Boston, Massachusetts, United States of America; Fukujuji Hospital, Tokyo, Japan; Instituto Nacional de Ciencias Médicas y de Nutrición “Salvador Zubirán”, Mexico, Mexico; University of Sassari, Sassari, Italy; National Jewish Health, Denver, Colorado, United States of America; Shaheed Beheshti Medical University, Tehran, Iran; Tropical Disease Foundation, Makati City, Philippines; University Medical Center Groningen, Groningen, The Netherlands; South African Medical Research Council, Pretoria, South Africa; University Medical Center Groningen, Groningen, The Netherlands; Instituto Nacional de Enfermedades Respiratorias, Mexico, Mexico; National Institute for Health Development, Tallinn, Estonia; Center for Infectious Diseases-California Department of Public Health, Sacramento, California, United States of America; Grantham Hospital, Hong Kong; 1 Bureau of Tuberculosis, New York, New York, United States of America; 2 A.G. Holley Hospital, Lantana, Florida, United States of America; 3 University of Toronto, Toronto, Canada; 4 Mayo Clinic, Rochester, Minnesota, United States of America; 5 Montreal Chest Institute, McGill University, Montreal, Canada; 6 The Dartmouth Center for Health Care Delivery Science, Hanover, New Hampshire, United States of America; 7 Harvard Medical School, Boston, Massachusetts, United States of America; 8 Partners in Health, Boston, Massachusetts, United States of America; 9 University of New Mexico School of Medicine, Albuquerque, New Mexico, United States of America; 10 WHO Collaborating Centre for TB and Lung Diseases, Care and Research Institute, Fondazione S. Maugeri, Tradate, Italy; 11 Denver Veterans Affair Medical Center, Denver, Colorado, United States of America; 12 Wan Fang Hospital, School of Medicine-Taipei Medical University, Taiwan; 13 Médecins Sans Frontières, Capetown, South Africa; 14 UC Davis School of Medicine, Davis, California, United States of America; 15 National TB Control Program, Hanoi, Vietnam; 16 International Union against Tuberculosis and Lung Disease, Paris, France; 17 World Health Organization, Geneva, Switzerland; 18 MRC Laboratories, Banjul, The Gambia; 19 California Department of Public Health, Sacramento, California, United States of America; 20 Instituto Nacional de Salud Pública, Mexico, Mexico; 21 Albert Einstein College of Medicine, Bronx, New York, United States of America; 22 Thailand MOPH & US CDC Collaboration, Bangkok, Thailand; 23 National Jewish Health, Denver, Colorado, United States of America; 24 University of California, San Francisco, San Francisco, United States of America; 25 Korea Cancer Center Hospital, Seoul, Korea; 26 Samsung Medical Center, Seoul, Korea; 27 South African Medical Research Council, Pretoria, South Africa; 28 Medical Clinic, Tuberculosis Center Borstel, Borstel, Germany; 29 University Medical Center Groningen, Groningen, The Netherlands; 30 Clinic of Tuberculosis and Lung Diseases, Riga, Latvia; 31 Tuberculosis and Chest Services, Hong Kong; 32 New York City Health and Mental Hygiene, New York, New York, United States of America; 33 Tomsk Oblast Tuberculosis Dispensary, Tomsk, Russia; 34 University of Washington, Seattle, Washington, United States of America; 35 City Road Medical Centre, London, United Kingdom; 36 Hospital F.J. Muñiz, Buenos Aires, Argentina; 37 TB Center, Seoul, Korea; 38 Imperial College London, London, United Kingdom; 39 Universidad Autonoma Madrid, Madrid, Spain; 40 Instituto de Salud del Estado de Aguascalientes, Mexico, Mexico; 41 Tropical Disease Foundation, Makati City, Philippines; 42 Instituto Nacional de Ciencias Médicas y de Nutrición “Salvador Zubirán”, Mexico, Mexico; 43 Bactériologie-Hygiène – UPMC, Paris, France; 44 Stellenbosch University, Stellenbosch, South Africa; 45 Brigham and Women's Hospital, Boston, Massachusetts, United States of America; 46 University of Ulsan College of Medicine, Seoul, Korea; 47 Fukujuji Hospital, Tokyo, Japan; 48 Instituto Nacional de Ciencias Médicas y de Nutrición “Salvador Zubirán”, Mexico, Mexico; 49 University of Sassari, Sassari, Italy; 50 Shaheed Beheshti Medical University, Tehran, Iran; 51 Instituto Nacional de Enfermedades Respiratorias, Mexico, Mexico; 52 National Institute for Health Development, Tallinn, Estonia; 53 Center for Infectious Diseases-California Department of Public Health, Sacramento, California, United States of America; 54 Grantham Hospital, Hong Kong; 55 Seoul National University College of Medicine, Seoul, Korea; Universidad Peruana Cayetano Heredia, Peru

## Abstract

**Background:**

Treatment of multidrug resistant tuberculosis (MDR-TB) is lengthy, toxic, expensive, and has generally poor outcomes. We undertook an individual patient data meta-analysis to assess the impact on outcomes of the type, number, and duration of drugs used to treat MDR-TB.

**Methods and Findings:**

Three recent systematic reviews were used to identify studies reporting treatment outcomes of microbiologically confirmed MDR-TB. Study authors were contacted to solicit individual patient data including clinical characteristics, treatment given, and outcomes. Random effects multivariable logistic meta-regression was used to estimate adjusted odds of treatment success. Adequate treatment and outcome data were provided for 9,153 patients with MDR-TB from 32 observational studies. Treatment success, compared to failure/relapse, was associated with use of: later generation quinolones, (adjusted odds ratio [aOR]: 2.5 [95% CI 1.1–6.0]), ofloxacin (aOR: 2.5 [1.6–3.9]), ethionamide or prothionamide (aOR: 1.7 [1.3–2.3]), use of four or more likely effective drugs in the initial intensive phase (aOR: 2.3 [1.3–3.9]), and three or more likely effective drugs in the continuation phase (aOR: 2.7 [1.7–4.1]). Similar results were seen for the association of treatment success compared to failure/relapse or death: later generation quinolones, (aOR: 2.7 [1.7–4.3]), ofloxacin (aOR: 2.3 [1.3–3.8]), ethionamide or prothionamide (aOR: 1.7 [1.4–2.1]), use of four or more likely effective drugs in the initial intensive phase (aOR: 2.7 [1.9–3.9]), and three or more likely effective drugs in the continuation phase (aOR: 4.5 [3.4–6.0]).

**Conclusions:**

In this individual patient data meta-analysis of observational data, improved MDR-TB treatment success and survival were associated with use of certain fluoroquinolones, ethionamide, or prothionamide, and greater total number of effective drugs. However, randomized trials are urgently needed to optimize MDR-TB treatment.

*Please see later in the article for the Editors' Summary*.

## Abstract 2

Dick Menzies and colleagues report findings from a collaborative, individual patient-level meta-analysis of treatment outcomes among patients with multidrug-resistant tuberculosis.

## Editors' Summary

**Background:**

In 2010, 8.8 million people developed tuberculosis—a contagious bacterial infection—and 1.4 million people died from the disease. *Mycobacterium tuberculosis*, the bacterium that causes tuberculosis, is spread in airborne droplets when people with the disease cough or sneeze and usually infects the lungs (pulmonary tuberculosis). The characteristic symptoms of tuberculosis are a persistent cough, weight loss, and night sweats. Tuberculosis can be cured by taking several powerful antibiotics regularly for at least 6 months. The standard treatment for tuberculosis comprises an initial intensive phase lasting 2 months during which four antibiotics are taken daily followed by a 4-month continuation phase during which two antibiotics are taken. However, global efforts to control tuberculosis are now being thwarted by the emergence of *M. tuberculosis* strains that are resistant to several antibiotics, including isoniazid and rifampicin, the two most powerful, first-line (standard) anti-tuberculosis drugs.

**Why Was This Study Done?:**

Although multi-drug resistant tuberculosis (MDR-TB) can be cured using second-line anti-tuberculosis drugs, these are more expensive and more toxic than first-line drugs and optimal treatment regimens for MDR-TB have not been determined. Notably, there have been no randomized controlled trials of treatments for MDR-TB. Such trials, which compare outcomes (cure, treatment failure, relapse, and death) among patients who have been randomly assigned to receive different treatments, are the best way to compare different anti-tuberculosis drug regimens. It is possible, however, to get useful information about the association of various treatments for MDR-TB with outcomes from observational studies using a statistical approach called “individual patient data meta-analysis.” In observational studies, because patients are not randomly assigned to different treatments, differences in outcomes between treatment groups may not be caused by the different drugs they receive but may be due to other differences between the groups. An individual patient data meta-analysis uses statistical methods to combine original patient data from several different studies. Here, the researchers use this approach to investigate the association of specific drugs, numbers of drugs and treatment duration with the clinical outcomes of patients with pulmonary MDR-TB.

**What Did the Researchers Do and Find?:**

The researchers used three recent systematic reviews (studies that use predefined criteria to identify all the research on a given topic) to identify studies reporting treatment outcomes of microbiologically confirmed MDR-TB. They obtained individual patient data from the authors of these studies and estimated adjusted odds (chances) of treatment success from the treatment and outcome data of 9,153 patients with MDR-TB provided by 32 centers. The use of later generation quinolones, ofloxacin, and ethionamide/prothionamide as part of multi-drug regimens were all associated with treatment success compared to failure, relapse or death, as were the use of four or more likely effective drugs (based on drug susceptibility testing of mycobacteria isolated from study participants) during the initial intensive treatment phase and the use of three or more likely effective drugs during the continuation phase. The researchers also report that among patients who did not die or stop treatment, the chances of treatment success increased with the duration of the initial treatment phase up to 7.1–8.5 months and with the total duration of treatment up to 18.6–21.5 months.

**What Do These Findings Mean?:**

These findings suggest that the use of specific drugs, the use of a greater number of effective drugs, and longer treatments may be associated with treatment success and the survival of patients with MDR-TR. However, these findings need to be interpreted with caution because of limitations in this study that may have affected the accuracy of its findings. For example, the researchers did not include all the studies they found through the systematic reviews in their meta-analysis (some authors did not respond to requests for individual patient data, for example), which may have introduced bias. Moreover, because the patients included in the meta-analysis were treated at 32 centers, there were many differences in their management, some of which may have affected the accuracy of the findings. Because of these and other limitations, the researchers note that, although their findings highlight several important questions about the treatment of MDR-TB, randomized controlled trials are urgently needed to determine the optimal treatment for MDR-TB.

**Additional Information:**

Please access these Web sites via the online version of this summary at http://dx.doi.org/10.1371/journal.pmed.1001300.

- The World Health Organization provides information on all aspects of tuberculosis, including MDR-TB; its guidelines for the programmatic management of drug-resistant tuberculosis are available
- The US Centers for Disease Control and Prevention has information about tuberculosis, including information on the treatment of tuberculosis and on MDR-TB
- The US National Institute of Allergy and Infectious Diseases also has information on all aspects of tuberculosis, including a drug-resistant tuberculosis visual tour
- MedlinePlus has links to further information about tuberculosis (in English and Spanish)
- TB & ME, a collaborative blogging project run by patients being treated for multidrug-resistant tuberculosis and Medecins sans Frontieres, provides information about MDR-TB and patient stories about treatment for MDR-TB
- The Tuberculosis Survival Project, which aims to raise awareness of tuberculosis and provide support for people with tuberculosis, also provides personal stories about treatment for tuberculosis

## Introduction

The increasing incidence of multidrug resistant tuberculosis (MDR-TB), defined as resistance to at least isoniazid and rifampin, is a major concern for TB control programs worldwide. MDR-TB treatment requires prolonged use of multiple second-line anti-TB drugs, which are more expensive and toxic than first-line drugs, yet less efficacious [1]. As a result of these problems, administration of MDR-TB treatment imposes substantial operational challenges in resource constrained settings. Further, the optimal composition and duration of MDR-TB treatment regimens is uncertain [1],[2].

Three systematic reviews have recently examined determinants of treatment outcomes in MDR-TB [3]–[5]. However, these three reviews identified no randomized trials, and the majority of the observational studies identified reported results with individualized treatment. There were considerable differences between studies in the diagnostic methods used, treatment regimens given, and clinical characteristics of the patient populations. As a result, these meta-analyses could only analyze pooled odds of treatment success associated with proportions of patients with specific clinical characteristics or receiving specific treatments. This approach has considerable limitations for a clinical problem of this complexity.

Even in the absence of randomized trials, an individual patient data meta-analysis of observational data offers potential benefits. Detailed patient level information can be used to estimate associations of treatment factors with outcomes, stratified by important covariates, within restricted sub-groups, or adjusted for covariates in meta-regression. We conducted an individual patient data meta-analysis using patient level data combined from different centers, using methods suggested by the Cochrane group [6]. We addressed several questions formulated by an expert committee of the World Health Organization (WHO) responsible for revision of guidelines for treatment of MDR-TB [7]. These questions included the impact of specific drugs, number of drugs, and duration of treatment on clinical outcomes of patients with pulmonary MDR-TB.

## Methods

### Selection of Studies

The studies considered for this individual patient data meta-analysis were identified from published original studies included in three recent systematic reviews of MDR-TB treatment outcomes [3]–[5]. All three reviews included studies published after 1970 that reported original data of treatment of patients with microbiologically confirmed MDR-TB. Additional specific criteria for this meta-analysis were: the study authors could be contacted and were willing to share their data; the cohort included at least 25 subjects treated for MDR-TB; and, at least treatment success, as defined below, was reported. Patients within these datasets were excluded if they had only extra-pulmonary TB, had extensive drug resistance (XDR-TB, as defined elsewhere [8]), or were missing treatment information.

### Data Sharing, Extraction, and Verification

Letters describing the meta-analysis were communicated to all corresponding authors of eligible studies. The McGill investigators signed formal data sharing agreements with all collaborating investigators regarding sharing of results, publications, and “ownership” of the data. This project was approved by the Research Ethics Board of the Montreal Chest Institute, McGill University Health Centre, and when deemed necessary by local ethics boards of originally approved studies.

Each author provided center-level information such as diagnostic laboratory methods, treatment regimen doses and supervision, and outcome definitions. Regimens were considered individualized if regimens were tailored to individual patients' characteristics such as prior therapy, or drug susceptibility testing (DST) results. Authors also provided de-identified patient level information including age, sex, HIV infection, site of disease, results of chest x-ray, acid fast bacilli (AFB) smear, culture, and DST for first and second-line drugs, drugs used and duration for initial and continuous phases of treatment, surgical resection, and outcomes, including adverse events that required a change in therapy. Treatment outcome definitions provided by each author were compared to the consensus definitions published by Laserson et al. [9], and rated as the same, closely similar, or not similar. These definitions are summarized at the top of Table S3. Relapse was any recurrence of disease after successful treatment completion, and was combined with failure for all analyses. For this analysis we considered the following as part of group 5 drugs: amoxicillin-clavulanate, macrolides (azithromycin, roxithromycin, and clarithromycin), clofazimine, thiacetazone, imipenem, linezolid, high dose isoniazid, and thioridazine.

Authors were contacted to request additional data and clarify variable definitions and coding. Variables from each original dataset were extracted, their meaning and coding verified, then mapped to a common set of variables for all patients. Hence datasets from each center had the same variables for all patients, but each dataset was kept distinct. As a final verification, the clinical characteristics of each study population were compared with the original published papers.

### Data Analysis

We considered three types of drug-exposure in our meta-analysis: (i) specific drugs administered (grouped as suggested by WHO [1]), (ii) duration of treatment regimen, and (iii) number of likely effective drugs used. Drugs were considered likely effective if susceptible on drug susceptibility testing, regardless of history of prior use. We estimated odds of treatment success (defined as treatment cure or completion) compared to one of three alternate outcomes: (i) treatment failure or relapse; (ii) treatment failure, relapse or *death*; and (iii) treatment failure, relapse, death or *default*. For duration of therapy, comparisons (ii) and (iii) were not analyzed because in studies with individualized therapy the planned duration was not recorded—only the actual duration, which was truncated by default or death during treatment.

We used random effects (random intercept and random slope) multi-variable logistic regression estimated via penalized quasi-likelihood (Proc Glimmix in SAS [10]) in order to estimate the adjusted odds and 95% CIs of treatment success associated with different treatment covariates [11]–[13]. As a sensitivity analysis, all models for primary analyses were also estimated using adaptive quadrature (QUAD) [14]. Patients were considered as clustered within studies and intercepts and slopes of the main exposure variables were allowed to vary across studies; this is to account for otherwise unmeasured inter-study differences in patient populations, as well as center-specific differences in data ascertainment, measurement, and other factors. The variance of the study specific intercepts (here the baseline log odds of success in each cohort) and slopes (here treatment efficacy) were interpreted to indicate how much these varied across the studies. We report the average estimate of effect across studies from these models and the estimated inter-study variability and standard deviation of that variance, as well as the variance of the intercept and the standard deviation of that variance.

Estimates of effect of each treatment parameter for each dataset were adjusted for five covariates: age, gender, HIV co-infection, extent of disease (considered extensive if AFB smear positive, or if AFB smear information was missing, then if there was cavitation on chest x-ray), and past history of TB treatment (a three level variable—no previous TB treatment, previous TB treatment with first-line drugs, and previous treatment with second-line drugs). Analyses were performed in all patients and in subgroups—stratified or restricted by important covariates. We tested for the interaction between previous treatment with second-line TB drugs and the number of drugs and duration of treatment in the intensive and continuation phases, respectively. In secondary analyses we included more than one treatment parameter (up to four drugs at once), and individual drugs with treatment duration. For the multivariable analyses only, missing values of these five clinical covariates were imputed using means of patients at the same center with non-missing information. Sex was missing in three patients, age was missing in 27, HIV was missing in 1,271(14%), history of past TB treatment missing in 443 (5%), history of past second-line drug use 758 (8%), and extent of disease information missing in 174 (2%). We assessed whether findings were altered when missing values were estimated using a different method of probabilistic imputation, in secondary analysis [15].

Additionally, heterogeneity was explored visually using Forest plots of study specific estimates, and estimated quantitatively via the *I* ^2^ and its associated 95% CI [13]. For these analyses, estimates of effect were calculated separately for each study, adjusting for relevant patient-level covariates, and pooled using conventional meta-analytic techniques. All analysis was performed using SAS, version 9.2 (SAS Institute).

## Results

### Selection of Studies and Description of the Study Population

**Figure 1:**
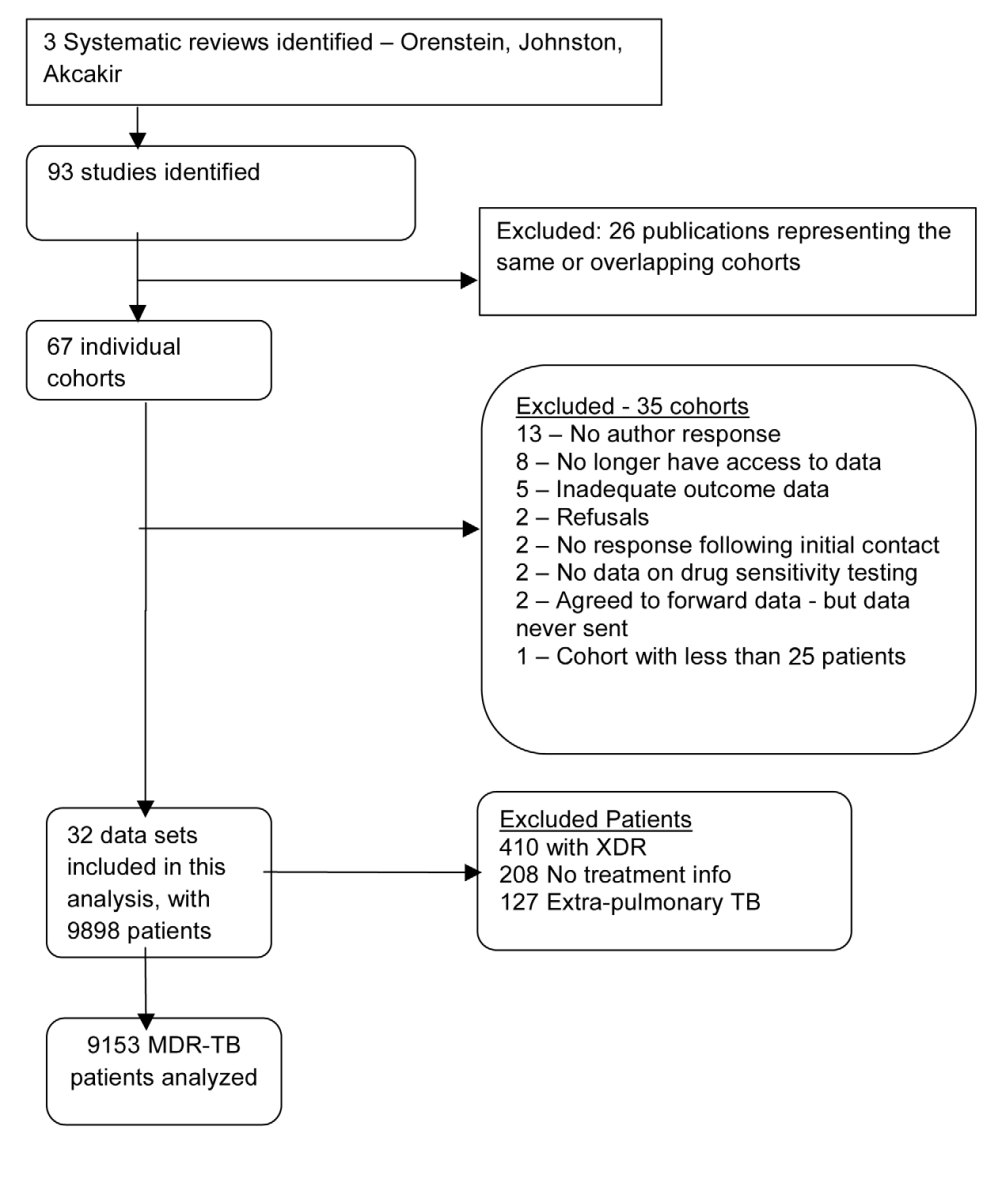
Study selection.

We identified 67 unique cohorts from the three previous systematic reviews of MDR-TB. Of these, 35 datasets were not used for reasons summarized in Figure 1, leaving 32 datasets with information on 9,898 patients [16]–[57]. From these we excluded 410 patients with XDR, 208 with inadequate treatment information, and 127 with solely extra-pulmonary TB, leaving 9,153 individual patients analyzed; their clinical characteristics and outcomes are summarized in Table 1.

**Table 1:** Clinical characteristics and treatment received of patients included in the analysis.

Demographic Characteristics	Data	Data	Data
Mean age in years (SD) (25 missing)	38.7 (13.6)	—	—
Male sex (%) (three missing)	6,280 (69%)	—	—
**Clinical characteristics**	**Yes**	**No**	**Missing**
AFB – smear positive (*n*, %)	6,012 (66%)	1,878 (21%)	1,263 (14%)
Cavities on x-ray (*n*, %)	4,723 (52%)	2,019 (22%)	2,411 (26%)
Extensive disease (*n*, %)	6,753 (74%)	2,226 (24%)	174 (2%)
HIV positive (*n*, %)	1,077 (12%)	6,805 (74%)	1,271 (14%)
Pulmonary TB only (*n*, %)	8,713 (96%)	232 (2%)	208 (2%)
Prior TB therapy (any)	6,683 (73%)	2,027 (22%)	443 (5%)
Prior therapy with second-line drugs	942 (10%)	7,455 (82%)	756 (8%)
**Resistance to other drugs**	**Sensitive**	**Resistant**	**Not tested**
Ethambutol (*n*, %)	2,736 (30%)	4,065 (44%)	2,352 (26%)
Pyrazinamide (*n*, %)	2,406 (26%)	2,443 (27%)	4,304 (47%)
Streptomycin (*n*, %)	2,454 (27%)	4,154 (45%)	2,545 (28%)
**Treatment received**			
Rifabutin (*n*, %)	130 (1.4%)	—	—
Ethambutol (*n*, %)	4,722 (52%)	—	—
Pyrazinamide (*n*, %)	6,571 (72%)	—	—
Ciprofloxacin (*n*, %)	986 (11%)	—	—
Ofloxacin (*n*, %)	6,489 (71%)	—	—
Later generation quinolones (*n*, %)	1,258 (14%)	—	—
Streptomycin (*n*, %)	1,326 (14%)	—	—
Kanamycin (*n*, %)	5,002 (55%)	—	—
Amikacin (*n*, %)	428 (5%)	—	—
Capreomycin (*n*, %)	1,757 (19%)	—	—
Ethionamide (*n*, %)	3,873 (42%)	—	—
Prothionamide (*n*, %)	3,709 (41%)	—	—
Cycloserine (*n*, %)	5,344 (58%)	—	—
Para-aminosalicylic acid (PAS) (*n*, %)	3,196 (33%)	—	—
One group 5 drug	2,115 (23%)	—	—
Two or more group 5 drugs	594 (7%)	—	—
**Outcomes (mutually exclusive)**			
Success (cure and treatment completed)	4,934 (54%)	—	—
Failure	645 (7%)	—	—
Relapse	87 (1%)	—	—
Default, transfer out, unknown	2,095 (23%)	—	—
Died during MDR-TB treatment	1,392 (15%)	—	—

Percentages are of all 9,153 patients. Extensive disease defined as AFB-smear positive, or cavities on chest x-ray if no information about AFB-smear. Prior TB therapy: defined as treatment with any, or second-line TB drugs for 1 mo or more. Later generation quinolones included levofloxacin, moxifloxacin, gatifloxacin, and sparfloxacin. Cycloserine included terizidone—a dimer of D-cycloserine given in some centers. Drugs analysed as group 5 included: amoxicillin-clavulanate, macrolides (azithromycin, roxithromycin, clarithromycin), clofazimine, thiacetazone, imipenem, linezolid, high dose INH, and thioridazine. Relapse ascertained in only 2,261 patients (14 cohorts).

SD, standard deviation.

**Figure 2:**
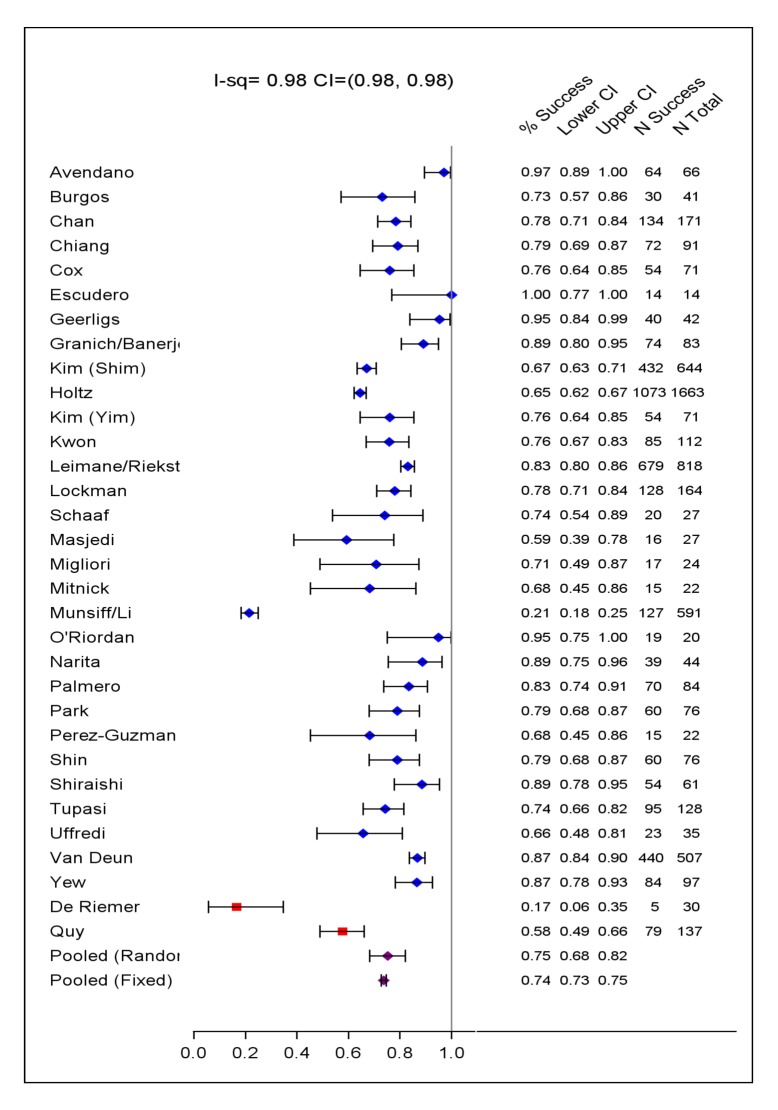
Treatment success versus failure and relapse and death. Crude treatment success versus failure or relapse or death by study with exact 95% CI, as well as number of subjects with success and number of subjects treated. Fixed and der Simonian and Laird random effects pooled estimates are given (purple dots). Two studies that used only first-line TB drugs are indicated by a red square.

**Figure 3:**
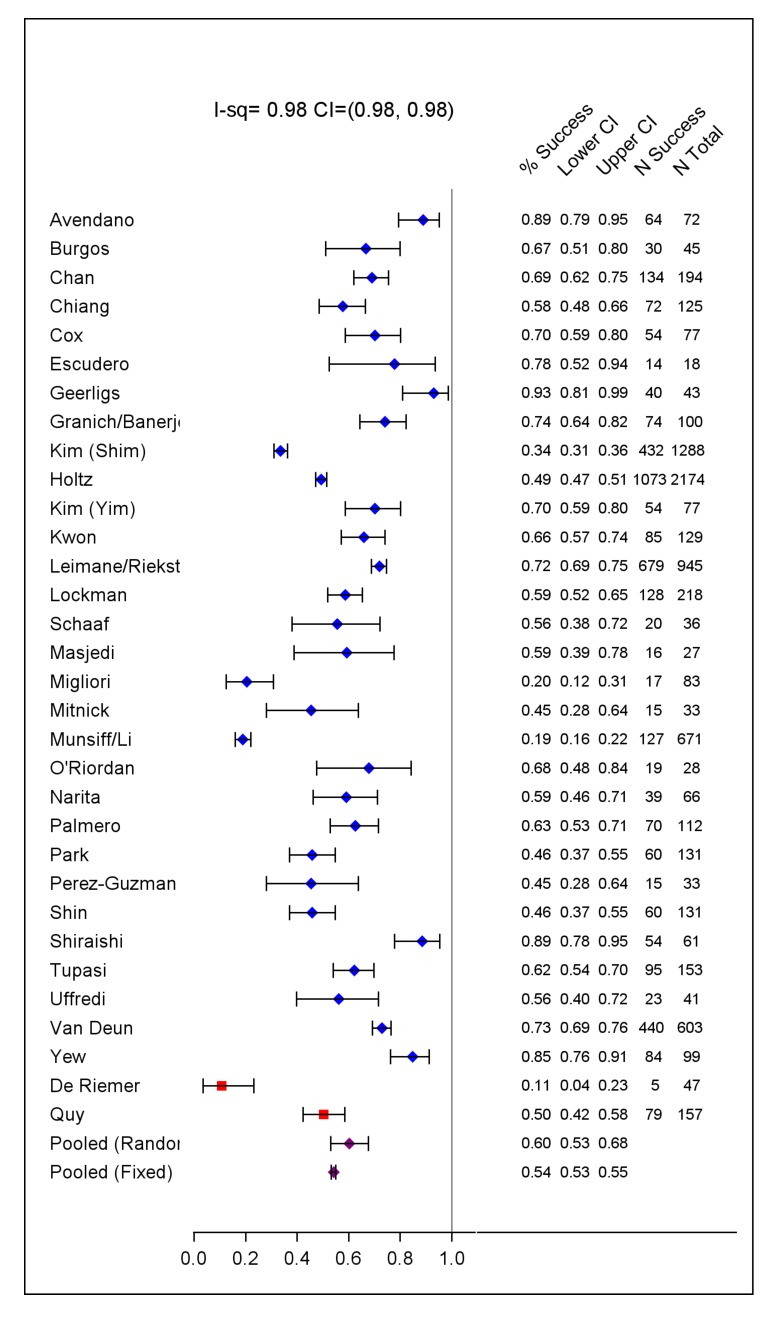
Treatment success versus failure and relapse and death. Fixed and der Simonian and Laird random effects pooled estimates are given (purple dots). Two studies that used only first-line TB drugs are indicated by a red square.

The included studies originated from 23 countries, from all WHO health regions. Final sample sizes included in the analysis ranged from 18 to 2,174 patients. In the supplement are summarized: study and center characteristics (Table S1a), excluded studies (Table S1b), doses of drugs commonly used (Table S2), and outcome definitions—the accepted standards and those used in each series (Table S3). Treatment was individualized in 26 studies with 5,985 patients, and standardized in six studies with 2,968 patients. A total of 200 patients in two centers received standardized regimens with first-line drugs only; all remaining patients received second-line drugs. In all but one study, the outcome definitions for treatment success and failure were judged the same or similar to the consensus definitions [9]. Overall 4,934 (54%) of patients were judged to have treatment success, 732 (8%) failed or relapsed, 1,392 (15%) died, and 2,095 (23%) defaulted (Table 1). Pooled treatment success, compared to failure/relapse/death, was 75% but varied widely between studies (Figure 2), while pooled success, compared to fail/relapse/death and default was only 54% (Figure 3). Adverse drug reactions that resulted in changed therapy occurred in 1932 (21%), and 499 (5%) patients underwent pulmonary resection surgery. Patients who died were significantly older, more likely to be HIV co-infected, with more extensive disease, and/or had prior therapy—with first-line and particularly with second-line TB drugs. Those who defaulted were significantly older, and more likely to have HIV co-infection (data not shown in tabular form).

### Treatment Correlates of Outcomes

As seen in Table 2, the use of later generation quinolones, or ofloxacin, or ethionamide/prothionamide, as part of multidrug regimens, was significantly associated with treatment success compared to failure or relapse. The use of these same drugs, as well as pyrazinamide or cycloserine were significantly associated with treatment success compared to failure, relapse, or death, while the use of later generation quinolones, ofloxacin, ethionamide/prothionamide or kanamycin were significantly associated with treatment success compared to failure, relapse, death, or default. Treatment outcomes of the 594 patients who received two or more group 5 drugs were significantly worse than the 2,115 patients who received one group 5 drug, whose outcomes were, in turn, worse than those of the 6,444 patients who received none of these drugs. Since this finding likely reflected confounding by indication for use of these drugs (i.e., group 5 drugs were used more often in patients with more severe disease or worse resistance), the analysis of the four most commonly used group 5 drugs—amoxicillin-clavulanate, macrolides, clofazimine, and thiacetazone—was restricted to patients who received only one group 5 drug. This revealed that none of these four drugs, compared to the others, was associated with significantly superior treatment outcomes. There was often significant heterogeneity in baseline odds of treatment success and in the treatment effect (as seen in the large variance relative to its standard deviation for the intercept and slope, respectively; these are provided in detail in Table S9.)

**Table 2:** Summary of association of use of individual drugs with treatment success.

Drug Used	*n* a	aOR^b^	Success Versus Failure/Relapse (95% CI)	*n* a	aOR^b^	Success Versus Failure/Relapse/Death (95% CI)	*n* a	aOR^b^	Success Versus Failure/Relapse/Death/Default (95% CI)
**Group 1 drugs**									
Pyrazinamide	3,985	1.2	(0.9–1.7)^c^	5,096	1.3	(1.1–1.6)^c^	6,571	1.1	(0.9–1.4)^c^
Ethambutol	2,819	0.9	(0.7–1.1)^d^	3,740	0.8	(0.7–0.9)^c^	4,719	0.9	(0.8–1.2)^c^
**Group 2: injectables**									
Kanamycin only	2,860			3,437			4,457		
Versus no injectable		1.1	(0.5–2.3)^e^		1.3	(0.7–2.6)^e^		1.3	(0.7–2.5)^e^
Versus capreomycin		1.3	(0.7–2.7)^c^		**1.6**	**(1.1–2.4)**		**1.3**	**(1.1–1.6)** d
Versus streptomycin		1.1	(0.6–2.2)^e^		1.0	(0.6–1.6)^e^		1.0	(0.8–1.3)^d^
Amikacin only	192			248			307		
Versus no injectable		1.5	(0.6–4.1)^d^		1.7	(0.8–3.3)		1.3	(0.5–3.6)^e^
Capreomycin only	769			940			1,127		
Versus no injectable		1.1	(0.5–2.6)^c^		1.3	(0.5–3.7)^e^		1.1	(0.4–3.2)^e^
**Group 3: quinolones**									
Later gen. quinolones	751			829			974		
Versus no quinolones		2.6	(0.6,10.5)^c^		**2.5**	**(1.0–5.9)**		**2.8**	**(1.3–6.1)** c
Versus ofloxacin		1.6	(0.5–5.3)^e^		**1.9**	**(1.0–3.6)**		**2.1**	**(1.2–3.9)** c
Versus ciprofloxacin		0.7	(0.1,3.8)^e^		1.5	(0.5–4.6)^e^		1.7	(0.6–4.9)^d^
Ofloxacin	3,832			4,577			6,102		
Versus no quinolones		**2.5**	**(1.8–3.9)** c		**2.5**	**(1.6–3.9)**		**2.0**	**(1.2–3.3)** e
Versus ciprofloxacin		1.1	(0.5–2.5)^c^		1.4	(0.7–2.6)		1.3	(0.7–2.5)^e^
Ciprofloxacin	335			553			644		
Versus no quinolones		1.5	(0.6–4.1)^d^		2.0	(0.8–5.2)		1.6	(0.6–4.3)^e^
**Group 4 drugs**									
Ethionamide/prothionamide	4,608	**1.6**	**(1.2–2.3)** c	5,594	**1.9**	**(1.6–2.2)** d	7,329	**1.7**	**(1.5–2.0)** d
Cycloserine/terizidone	3,547	1.1	(0.8–1.7)^c^	4,194	1.5	(1.0–2.3)	5,358	1.5	(0.9–2.2)^e^
PAS	2,459	1.0	(0.8–1.3)	2,860	1.0	(1.0–1.4)^d^	3,712	1.2	(1.0–1.5)^c^
Group 5 drugs									
Any 1 group 5 versus none	1,538	**0.6**	**(0.4–0.9)** c	1,725	**0.7**	**(0.6–0.8)** c	2,029	1.0	(0.8–1.2)^d^
2+ group 5 versus one group 5	447	**0.4**	**(0.3–0.6)** c	574	**0.5**	**(0.3–0.6)** c	654	**0.7**	**(0.5–0.9)** d
Amox.-clavulanate only^f^	232	1.0	(0.4–2.5)^c^	255	1.2	(0.6–2.6)^c^	290	1.4	(0.8–2.5)^c^
Clofazimine only^f^	651	2.7	(0.6–12.1)^e^	764	2.3	(0.4–12.4)^e^	896	1.0	(0.5–2.1)^e^
Macrolide only^f^	333	**0.4**	**(0.3–0.6)** d	396	**0.5**	**(0.4–0.7)** c	459	0.8	(0.6–1.1)^c^
Thiacetazone only^f^	554	0.8	(0.5–1.5)^d^	576	1.0	(0.6–1.7)^d^	668	1.0	(0.7–1.4)^d^

Bold, estimates are significantly different from the reference group.

a *n* shown, the number of patients that received the drug in question and were included in the specific analysis.

b aOR for use of drug, with non-use as the reference category. Adjusted for age, sex, HIV, past TB treatment, past MDR treatment (treatment for more than 1 mo with two or more second-line drugs), and extent of disease. Missing information was imputed for the following parameters in the following number of patients: Sex was missing in three, age was missing in 27, HIV was missing in 1,271 (14%), history of past TB treatment missing in 443 (5%), history of past second-line drug use 758 (8%), and extent of disease information missing in 174 (2%).

c Variance of the random intercepts and slopes low, so heterogeneity not likely to be important.

d Variance of the random intercepts and slopes could not be estimated.

e Variance of the random intercepts and slopes high, so heterogeneity likely important.

f Group 5 individual drugs: Analysis restricted to patients who received only one group 5 drug. Each single drug comparison made between patients who received only that group 5 agent with patients who received any other single group 5 drug. Drugs included in this analysis as group 5 drugs were: amoxicillin-clavulanate, macrolides (azithromycin, roxithromycin, and clarithromycin), clofazimine, thiacetazone, imipenem, linezolid, high dose INH, and thioridazine. Later generation quinolones included levofloxacin, moxifloxacin, gatifloxacin, and sparfloxacin. Cycloserine included terizidone—a dimer of D-cycloserine given in some centers.

Patients with prior treatment with second-line drugs were significantly less likely to have HIV co-infection, but were more likely to have cavitary disease, and strains with resistance to other first-line drugs (Table S4). In these patients the odds of treatment success with the individual drugs were similar, although CIs were broad (Table S5).

As shown in Table 3, compared to use of three or fewer likely effective drugs in the initial intensive phase, the odds of success were significantly greater with use of four drugs, and were very similar with use of five, six, or more drugs (Table 3). In the continuation phase, compared to use of two or fewer likely effective drugs, use of three drugs was associated with significantly superior odds of success, which were similar to the odds of success with use of four, or five or more likely effective drugs (Table 3). Fewer patients were included in these analyses because only a subset of studies provided information on the number of likely effective drugs used in the initial intensive phase (18 studies) or the continuation phase (15 studies). In patients with prior treatment with second-line drugs the maximal odds of success was seen with five likely effective drugs in the initial intensive phase (Table 4), and five drugs in the continuation phase (Table 4). There was substantial heterogeneity and a statistically significant interaction between prior treatment with second-line drugs and number of drugs used in the continuation phase (*p = *0.01), and in the initial intensive phase (*p = *0.05) (Tables S10). In further exploratory analyses, there was no association between the number of drugs used in the initial phase with default, but default was more frequent in patients who received more drugs in the continuation phase (Table S11).

**Table 3:** Association of number of likely effective drugs with treatment success—during different phases of treatment.

*n* Likely Effective Drugs – All Patients – Three Analyses	All Patients – Success Versus Fail/Relapse		All Patients – Success Versus Fail/Relapse/Death		All Patients – Success Versus Fail/Relapse/Death/Default	
	*n*	aOR (95% CI)	*n*	aOR (95% CI)	*n*	aOR (95% CI)
Initial intensive phase						
0–2	118	1.0 (reference)	277	1.0 (reference)	322	1.0 (reference)
3	161	1.1 (0.5–2.4)^a^	250	1.7 (1.2–2.5)^a^	316	1.2 (0.8–1.8)^a^
4	468	**2.0 (1.1–3.6)** a	542	**2.7 (1.9–3.9)** a	671	**1.9 (1.3–2.9)** a
5	814	**2.0 (1.1–3.6)** a	900	**2.8 (1.7–4.6)** a	1,114	**1.9 (1.2–3.0)** a
6+	811	**2.4 (1.0–5.4)** a	977	**2.1 (1.4–3.1)** a	1,185	**1.4 (1.0–2.1)** a
Continuation phase						
0–2	254	1.0 (reference)	531	1.0 (reference)	633	1.0 (reference)
3	552	**2.5 (1.6–4.0)** b	635	**5.7 (3.4–9.7)** a	759	**4.9 (2.7–8.7)** a
4	598	**2.8 (1.6–4.9)** a	663	**5.7 (3.2–10.0)** a	779	**4.2 (2.6–6.7)** a
5+	560	**2.0 (0.9–4.2)** a	608	**7.0 (5.1–9.7)** b	656	**4.9 (2.5–9.5)** a

Likely effective, drugs to which isolate susceptible in laboratory testing. *n*, number of patients in subgroup of interest. aOR, adjusted for age, sex, HIV, past TB treatment, past MDR treatment (treatment for more than 1 mo with two or more second-line drugs), and extent of disease. Missing information was imputed for the following parameters in the following number of patients: Sex was missing in three, age was missing in 27, HIV was missing in 1,271(14%), history of past TB treatment missing in 443 (5%), history of past second-line drug use 758 (8%), and extent of disease information missing in 174 (2%). Success, defined as cure or treatment completion; see Methods for definitions. Initial intensive phase, period when injectable given. Continuation phase, period when no injectable given. Only 18 studies provided information regarding drug susceptibility testing and the number of drugs in the initial phase, while only 15 of these described the number of drugs in the continuation phase. Bold, estimates are significantly different from the reference group.

a Variance of the random intercepts and slopes was low—so heterogeneity not likely to be important.

b Variance of the random intercepts and slopes could not be estimated.

**Table 4:** Effect of previous treatment on association of number of likely effective drugs with treatment success—during different phases of treatment.

*n* Likely Effective Drugs – Patients Stratified by Treatment History	All Patients Success Versus Fail/Relapse/Death			No Prior MDR Treatment Success Versus Fail/Relapse/Death			Prior MDR Treatment Success Versus Fail/Relapse/Death		
	*n*	aOR	(95% CI)	*n*	aOR	(95% CI)	*n*	aOR	(95% CI)
**Initial phase**									
**0–2**	277	1.0	(reference)	246	**1.0**	**(reference)**	**6**	**1.0**	**(reference)**
**3**	250	**1.7**	**(1.2–2.5)**	**186**	**2.3**	**(1.4–2.9)**	**16**		
**4**	542	**2.7**	**(1.9–3.9)**	**385**	**3.4**	**(2.2–5.2)**	**66**	1.4	(0.5–3.8)
**5**	900	**2.8**	**(1.7–4.6)**	**650**	**2.6**	**(1.7–4.0)**	**111**	**3.4**	**(1.2–9.8)**
**6+**	**977**	**2.1**	**(1.4–3.1)**	**515**	**3.1**	**(2.0–4.9)**	**327**	**1.6**	**(0.3–9.1)**
**Continuation phase**									
**0–2**	531	1.0	(reference)	467	1.0	(reference)	32	1.0	(reference)
**3**	**635**	**5.7**	**(3.4–9.7)**	**532**	**5.5**	**(2.2–13.6)**	**46**	**5.5**	**(2.3–13.1)**
**4**	**663**	**5.7**	**(3.2–10.0)**	**430**	**3.3**	**(1.8–6.3)**	**89**	**9.1**	**(4.4–18.8)**
**5+**	**608**	**7.0**	**(5.1–9.7)**	**265**	**4.6**	**(1.5–14.0)**	**211**	**13.7**	**(8.2–23.0)**

Likely effective, drugs to which isolate susceptible in laboratory testing. *n*, number of patients in subgroup of interest. aOR, adjustment described in footnotes for Table 3. Success, defined as cure or treatment completion; see Methods for definitions. Initial intensive phase, period when injectable given. Continuation phase, period when no injectable given. Only 18 studies provided information regarding drug susceptibility testing and the number of drugs in the initial phase, while only 15 of these described the number of drugs in the continuation phase. Bold, estimates are significantly different from the reference group.

**Figure 4:**
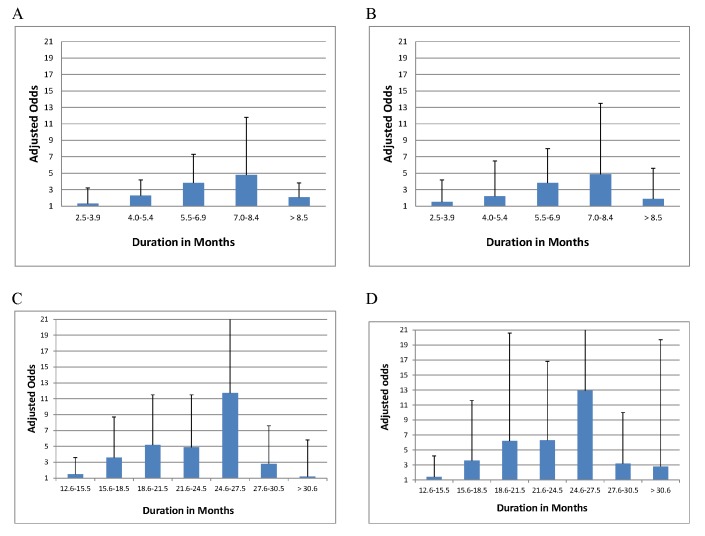
Association of treatment success with duration (adjusted odds and upper bound of CI shown). (A) Duration of initial intensive phase in all patients (reference group 1.0–2.5 mo). (B) Duration of initial intensive phase—restricted to patients not previously treated with second-line drugs (reference group 1.0–2.5 mo). (C) Total duration of therapy in all patients (reference group is 6.0–12.5 mo). Patients receiving therapy for less than 6 or more than 36 mo excluded from analysis. Note: For duration of 24.6–27.5 mo the upper limit of the CI was 30.2. This is truncated at 21. (D) Total duration of therapy—analysis restricted to patients not previously treated with second-line drugs (reference group is 6.0–12.5 mo. Patients receiving therapy for less than 6 or more than 36 mo excluded from analysis). Note: For duration of 24.6–27.5 mo, the upper limit of the CI was 56.5. This is truncated at 21.

Among those who did not die or default, odds of treatment success increased with longer duration of the initial intensive phase up to the duration of 7.0 to 8.4 mo (Table 5), although CIs were wide for each interval estimate (Figure 4), and variance estimates were also high (Tables S12). There was no statistically significant interaction between intensive phase or total treatment duration and prior treatment with second-line drugs. Similarly odds of success increased progressively with longer total duration of therapy up to 24.6–27.5 mo duration. In patients with prior second-line drug therapy the maximal odds of success was seen with total duration of 27.6–30.5 mo, although there were very few patients in each strata, and CIs were correspondingly wide. As seen in Tables S6–S8, additional secondary analyses of optimal duration revealed similar results for: analyses with adjustment for use of four drugs, in addition to adjustment for the five clinical covariates used for all other models (Table S6A), patients who received only one injectable and experienced no injectable-related adverse events (Table S6B), or patients who received regimens containing at least one second-line drug (Table S6C). In patients who received later generation quinolones the maximal odds of success was seen with a shorter duration of total therapy (Table S6D), although CIs were very wide, and included 1.0. There was a significant trend toward more frequent history of prior second-line drug treatment, but lower HIV prevalence with longer duration of initial intensive phase (Table S8a) and total duration (Table S8b) and somewhat greater prevalence of resistance to pyrazinamide, ethambutol, fluoroquinolones, or second-line injectables with longer initial intensive or total duration.

**Table 5:** Association of duration of treatment with success versus failure/relapse—patients grouped by treatment history.

Duration of Treatment (mo) Intercept	All Patients		No Prior Second-Line Drug Treatment		Prior Second-Line Drug Treatment	
	*n*	aOR (95% CI)	*n*	aOR (95% CI)	*n*	aOR (95% CI)
Initial						
1–2.4	308	1.0 (reference)	271	1.0 (reference)	6	1.0 (reference)
2.5–3.9	1,406	1.3 (0.5–3.2)^a^	1,298	1.5 (0.6–4.2)^b^	23	4.2 (0.5–34.3)^b^
4.0–5.4	481	**2.3 (1.2–4.2)** b	418	2.2 (0.8–6.5)^b^	15	10.9 (1.0–117.8)^b^
5.5–6.9	377	**3.8 (2.0–7.3)** b	314	**3.8 (1.8–8.0)** b	26	**47.2 (3.0–746.1)** b
7.0–8.4	172	**4.8 (1.9–11.8)** b	124	**4.9 (1.8–13.5)** b	21	**—**
8.5–20	792	**2.1 (1.2–3.8)** b	517	**1.9 (0.6–5.6)** a	228	**26.3 (3.8–183.9)** b
Total						
6.0–12.5	778	1.0 (reference)	681	1.0 (reference)	33	1.0 (reference)
12.6–15.5	419	1.5 (0.6–3.6)^a^	321	1.4 (0.5–4.2)^a^	34	0.4 (0.2–1.1)^b^
15.6–18.5	1,700	**3.6 (1.5–8.7)** a	1,527	**3.6 (1.1–11.6)** a	51	2.2 (0.7–6.8)^b^
18.6–21.5	655	**5.2 (2.0–11.5)** a	34	**6.2 (1.8–20.6)** a	40	1.6 (0.6–4.5)^b^
21.6–24.5	553	**4.9 (2.1–11.5)** b	400	**6.3 (2.3–16.8)** b	105	**6.5 (2.2–19.7)** b
24.6–27.5	313	**11.7 (4.5–30.2)** b	170	**12.9 (3.0–56.5)** b	104	**8.1 (2.1–31.4)** b
27.6–30.5	160	**2.8 (1.0–7.6)** b	89	**3.2 (1.0–10.0)** b	53	**13.6 (1.6–114.1)** b
30.6–36	89	1.2 (0.2–5.8)^a^	36	2.8 (0.4–19.7)^b^	38	2.0 (0.6–7.3)^b^

*n*, number of patients in subgroup of interest. aOR, adjusted odds ratios—adjustment described in footnotes for Table 3. Success, defined as cure or treatment completion and is compared to failure or relapse (see methods for definitions). Other outcomes of death and default not assessed in this analysis because in some datasets shorter duration that was directly due to death or default could not be identified. Past treatment, prior MDR means past treatment for more than 1 mo with two or more second-line drugs. No prior MDR includes all other treatment history.

a Variance of the random intercepts and slopes high—so heterogeneity likely important. (See Tables S1–S12 for actual values).

b Variance of the random intercepts and slopes low—so heterogeneity not likely to be important. (See also Tables S1–S12 for actual values).

As a sensitivity analysis, all analyses reported in Tables 2–4 were repeated using random effects logistic regression estimated via adaptive quadrature. Results, except where indicated in the tables, were very similar to those estimated using penalized quasi-likelihood. In addition analyses using probabilistic imputation for missing values produced very similar results as results using the imputation methods described above.

## Discussion

To our knowledge this is the largest combined analysis of treatment of MDR-TB, and the first individual patient data meta-analysis of treatment outcomes in drug resistant TB. With the detailed individual clinical information for 9,153 patients it was possible to use stratified, restricted, and/or multivariable analyses to control for differences in treatment regimens, drug resistance patterns, prior treatment histories, and other patient characteristics such as HIV co-infection. Overall treatment results were poor—treatment success was achieved in only slightly more than half of all patients. Treatment success was significantly associated with specific durations, number of likely effective drugs for the initial intensive and continuation phases of therapy, and with use of later generation quinolones (levofloxacin, moxifloxacin, gatifloxacin, and sparfloxacin), ofloxacin or ethionamide/prothionamide. These results helped to inform the forthcoming revised MDR-TB treatment guidelines of WHO, and should be useful in planning therapy for individual patients.

We suggest cautious interpretation of these results in light of a number of important limitations. First, we included only 32 studies out of a possible 67 series that had reported outcomes of MDR-TB treatment and were identified in three systematic reviews. This selection may have introduced some bias, although as seen in Table S1b, the characteristics and outcomes of patients in the included and excluded studies were similar. Second, we included two studies in which most patients received only first-line drugs; this may seem obviously inadequate, but earlier reports supported such treatment [58]. However, only 200 patients were treated in this way, and findings were not changed when they were excluded from analyses. Third, all the data included in this review were derived from observational studies, most of which utilized individualized regimens; this may have introduced bias if certain drugs, or combinations and durations of drugs were preferentially used for patients with more extensive drug resistance, more severe disease, or worse co-morbidities. An example of this selection bias was the finding that use of any group 5 drug was associated with worse outcomes, particularly use of two or more group 5 drugs. As recommended [59], we controlled this potential confounding by restricting the analysis to patients who received only one of the group 5 agents. Fourth, the effects of individual drugs may have been difficult to detect because of the multidrug composition of all regimens used. Furthermore certain drugs such as amikacin were used less often—limiting power—while others, such as linezolid or high dose isoniazid were used so infrequently they could not be analyzed. The small size of the “no-injectable” group may have limited power to detect an impact of the injectables, perhaps explaining the observed modest effect.

Relapse was ascertained in only 14 studies, which could result in an over-estimate of treatment success. As seen in Table S7, success rates were non-significantly lower in centers that did measure relapse. Finally, since patients included in this analysis were treated in more than 32 different centers (some studies involved multiple centers), management differed considerably in terms of use of hospitalization, response to adverse events, use of adjunctive surgery, or directly observed therapy, and the resources and adherence support offered to patients. These unmeasured inter-center differences could have resulted in bias. For example in centers that could afford to use later generation fluoro-quinolones, which are more expensive, there may have been greater resources to enhance care in other ways. However, the random effects meta-analytic approach provided some control for these center-level effects.

This analysis suggests that it would be appropriate to use at least four likely effective drugs in the initial intensive phase and at least three likely effective drugs in the continuation phase. However, it is important to underline that this analysis was restricted to cohorts of patients in whom drug susceptibility testing was routinely performed. These results may not apply when standardized regimens are used without routine drug susceptibility testing. We had to base analysis of likely effective drugs on drug susceptibility testing only, because of limited information on the specific drug regimen for many of the previously treated patients. Hence caution is warranted given the well-known limitations of drug susceptibility testing for many of the drugs used, since prior use of these drugs may increase the likelihood of resistance, even if the laboratory result indicates susceptibility.

The highest odds of success were associated with duration of the initial intensive phase of 7–8.4 mo, and with a total duration of 18–20 mo. However, particular caution should be used for the interpretation of these results. First we did not have data on duration of therapy with individual drugs, only the different phases of treatment. Second, duration of therapy was individualized for most patients, based upon severity of disease, prior therapy, drug resistance patterns, response to therapy, and timing of sputum conversion. Hence duration of treatment may have been prolonged in patients with worse disease—as suggested in Tables S7a and S7b. This could have accounted for the finding that treatment success was less with very long treatment durations, although would not be expected to lead to the finding that the odds of success increased progressively with each interval of initial intensive phase therapy up to 7–8.5 mo (in fact would be expected to have the opposite effect). Third, we had limited information on microbiologic responses, and so could not analyze the effect of duration after microbiologic conversion—a cornerstone of current recommendations (although the published evidence for the relationship between sputum conversion and long term outcomes in MDR-TB is sparse). As a result conclusions must be cautious regarding the optimal duration of therapy, which must balance the burden of prolonged therapy on patients and health systems, with the possible benefits demonstrated in this analysis. A recent report from Bangladesh in which treatment success rates were high with much shorter treatment [54], underscores the need for appropriately framed randomized trials to address this issue [60].

Despite these limitations there were a number of important strengths. A large number of centers, from many different regions of the world, contributed clinical information on a large number of individual patients, allowing a detailed and comprehensive analysis. There was substantial variation in treatment given by different centers, only partially explained by patients' characteristics. In some centers this variation reflected availability of medications, but in other centers this likely reflected individual providers' preferences. This substantial variation in treatment approach would have been much less likely in patients treated at a single center, and enhanced our ability to assess the independent effect of treatment factors on patient outcomes.

### Conclusions

This individual patient data meta-analysis of 9,153 patients suggests that treatment of MDR-TB should include a later generation quinolone, and ethionamide or prothionamide. In patients who have not received second-line drugs before, the optimal number of likely effective drugs appears to be at least four in the initial intensive phase, and at least three in the continuation phase. The duration of therapy associated with highest odds of success was 7–8.5 mo for the initial intensive phase, and 25–27 mo for total duration. In view of the serious limitations of this observational data, these findings should be considered to have highlighted several important questions for future clinical trials. These questions include the role and choice of injectables, the optimal duration of an injectable and total therapy, and the potential value of later generation quinolones as well as certain group 4 and group 5 drugs. Randomized trials are urgently needed to address these questions and determine the optimal treatment regimens for MDR-TB patients.

## Supporting Information

Alternative Language Abstract S1 **Chinese translation of the abstract by C-YC and Yuhong Liu.**
(DOCX)

Alternative Language Abstract S2 **French translation of the abstract by JR.**
(DOCX)

Alternative Language Abstract S3 **German translation of the abstract by CL.**
(DOCX)

Alternative Language Abstract S4 **Italian translation of the abstract by GS and GBM.**
(DOCX)

Alternative Language Abstract S5 **Korean translation of the abstract by T-SS, W-JK, and J-JY.**
(DOCX)

Alternative Language Abstract S6 **Spanish translation of the abstract by DP, MLGG, JS-O, AP-d-L, MHV, and CP-G.**
(DOCX)

Alternative Language Abstract S7 **Japanese translation of the abstract by MN and YS.**
(DOCX)

Alternative Language Abstract S8 **Russian translation of the abstract by DM.**
(DOC)

Table S1 (a) Overview of settings of 32 studies included in individual patient data meta-analysis of MDR-TB supplemental tables (for on-line supplement—references for included studies are found in main text. (b) Overview of 35 studies excluded from individual patient data meta-analysis of MDR-TB.
(DOC)

Table S2 **Dosages of drugs used for MDR-TB treatment at sites of studies included in individual patient data meta-analysis on MDR-TB supplemental tables (for on-line supplement—references for included studies are found in main text.**
(DOC)

Table S3 **Summary of treatment outcome definitions used in studies included in individual patient data meta-analysis.**
(DOC)

Table S4 **Characteristics of patients associated with history of prior TB therapy.**
(DOC)

Table S5 **Association of Individual drugs with treatment success (compared to failure/relapse)—stratified by history of previous treatment.**
(DOC)

Table S6 **Secondary analyses to assess impact of covariates on duration of therapy.** (A) Duration analysis also adjusted for use of four drugs. (B) Analysis restricted: excluded patients who received two or more injectables, and/or had a serious adverse event to an injectable (204 patients in two studies treated with first-line drugs also excluded). (C) Restricted analyses: use of second-line drugs only. (D) Stratified analysis: by use of later generation quinolones: 204 patients receiving first-line drugs only excluded.
(DOC)

Table S7 **Pooled outcomes—studies stratified by study level factors (event rates pooled across studies—study level random effects meta-analysis).**
(DOC)

Table S8 (a) Assessment of potential confounding of clinical characteristics and drug resistance with initial duration (only patients analyzed for success versus fail/relapse). (B) Assessment of potential confounding of clinical characteristics with total duration of therapy (only patients analyzed for success versus fail/relapse).
(DOC)

Table S9 **Summary of variance of estimates—individual drugs with treatment success.**
(DOC)

Table S10 **Summary of variance of estimates—likely effective drugs with treatment success—during different phases of treatment.**
(DOC)

Table S11 **Association of number of drugs used and treatment success compared to default only.**
(DOC)

Table S12 **Summary of variance of estimates—duration of treatment with success versus failure/relapse—patients grouped by treatment history.**
(DOC)

## Abbreviations

AFB: acid fast bacilli
aOR: adjusted odds ratio
MDR-TB: multidrug resistant tuberculosis
