# Correction: Multidrug Resistant Pulmonary Tuberculosis Treatment Regimens and Patient Outcomes: An Individual Patient Data Meta-analysis of 9,153 Patients

**DOI:** 10.1371/annotation/230240bc-bcf3-46b2-9b21-2e6e584f7333

**Published:** 2012-09-14

**Authors:** Shama D. Ahuja, David Ashkin, Monika Avendano, Rita Banerjee, Melissa Bauer, Jamie N. Bayona, Mercedes C. Becerra, Andrea Benedetti, Marcos Burgos, Rosella Centis, Eward D. Chan, Chen-Yuan Chiang, Helen Cox, Lia D'Ambrosio, Kathy DeRiemer, Nguyen Huy Dung, Donald Enarson, Dennis Falzon, Katherine Flanagan, Jennifer Flood, Maria L. Garcia-Garcia, Neel Gandhi, Reuben M. Granich, Maria G. Hollm-Delgado, Timothy H. Holtz, Michael D. Iseman, Leah G. Jarlsberg, Salmaan Keshavjee, Hye-Ryoun Kim, Won-Jung Koh, Joey Lancaster, Christophe Lange, Wiel C. M. de Lange, Vaira Leimane, Chi Chiu Leung, Jiehui Li, Dick Menzies, Giovanni B. Migliori, Sergey P. Mishustin, Carole D. Mitnick, Masa Narita, Philly O'Riordan, Madhukar Pai, Domingo Palmero, Seung-kyu Park, Geoffrey Pasvol, Jose Peña, Carlos Pérez-Guzmán, Maria I. D. Quelapio, Alfredo Ponce-de-Leon, Vija Riekstina, Jerome Robert, Sarah Royce, H. Simon Schaaf, Kwonjune J. Seung, Lena Shah, Tae Sun Shim, Sonya S. Shin, Yuji Shiraishi, José Sifuentes-Osornio, Giovanni Sotgiu, Matthew J. Strand, Payam Tabarsi, Thelma E. Tupasi, Robert van Altena, Martie Van der Walt, Tjip S. Van der Werf, Mario H. Vargas, Pirett Viiklepp, Janice Westenhouse, Wing Wai Yew, Jae-Joon Yim, Collaborative Group for Meta-Analysis of Individual Patient Data in MDR-TB

The eleventh author's name is misspelled. The correct spelling is: Edward D. Chan.

