# Supplementary material for: Multidrug Resistant Pulmonary Tuberculosis Treatment Regimens and Patient Outcomes: An Individual Patient Data Meta-analysis of 9,153 Patients
Source: PLoS Med. 2012 Aug 28;9(8):e1001300. doi: 10.1371/journal.pmed.1001300 (PMC3429397; doi:10.1371/journal.pmed.1001300)
Supplement: Alternative Language Abstract S1 — Chinese translation of the abstract by C-YC and Yuhong Liu. (DOCX) [file pmed.1001300.s001.docx]

**Alternative Language Abstract 1: Chinese**

**Translation of the abstract “Multidrug-resistant pulmonary tuberculosis treatment regimens and patient outcomes: an individual patient data meta-analysis of 9153 patients.” Into Chinese by authors Chen-Yuan CHIANG, Yuhong Liu**

耐多药肺结核治疗方案和病人转归：9153例病例个案资料的荟萃分析

耐多药结核病人个案资料荟萃分析协作小组

摘要：

背景：耐多药结核病（MDR-TB）的治疗是漫长、有毒性、昂贵的，并且通常效果不佳。我们进行了一项病例个案资料的荟萃分析，评估用于MDR-TB治疗的药物种类、药物数量和用药时间对于转归的影响。

方法：利用最近的三篇系统综述找寻关于细菌学确定的MDR-TB病人的治疗转归的研究报告。联络研究的作者，请求提供关于病人临床特征、所给予的治疗、以及转归的个案信息。使用“随机效应多元回归模型”估计病人治疗成功的调整后比值比。

结果：32个观察性研究提供了9153例MDR-TB病人治疗和转归的足够信息。与治疗失败/复发相比，治疗成功与下列药物使用相关：新代喹诺酮类药物（调整后比值比(aOR)：2.5[95%可信区间：1.1, 6.0]），氧氟沙星 (aOR：2.5 [1.6, 3.9]), 乙硫异烟胺或丙硫异烟胺(aOR: 1.7 [1.3, 2.3]),最初强化期使用四种或四种以上可能有效的药物(aOR: 2.3 [1.3, 3.9]), 继续期使用三种或三种以上可能有效的药物 (aOR: 2.7 [1.7, 4.1])。与治疗失败/复发或死亡相比较，结果相似，治疗成功与下列药物的使用相关：新代喹诺酮类药物(aOR: 2.7 [1.7, 4.3]), 氧氟沙星(aOR: 2.3 [1.3, 3.8]), 乙硫异烟胺或丙硫异烟胺(aOR: 1.7 [1.4, 2.1]), 最初强化期使用四种或四种以上可能有效的药物(aOR: 2.7 [1.9, 3.9]), 继续期使用三种或三种以上可能有效的药物(aOR: 4.5 [3.4, 6.0])。

结论：在本次病例个案信息的荟萃分析中，耐多药结核病治疗成功率和生存率的改善与使用某些氟喹诺酮类药物、乙硫异烟胺或丙硫异烟胺、以及应用多种有效药物治疗相关。随机对照试验急需开展，以优化耐多药结核的治疗。

翻译:刘宇红
