# Supplementary material for: Multidrug Resistant Pulmonary Tuberculosis Treatment Regimens and Patient Outcomes: An Individual Patient Data Meta-analysis of 9,153 Patients
Source: PLoS Med. 2012 Aug 28;9(8):e1001300. doi: 10.1371/journal.pmed.1001300 (PMC3429397; doi:10.1371/journal.pmed.1001300)
Supplement: Alternative Language Abstract S2 — French translation of the abstract by JR. (DOCX) [file pmed.1001300.s002.docx]

**Alternative Language Abstract 2: French**

**Translation of the abstract Multidrug-resistant pulmonary tuberculosis treatment regimens and patient outcomes: an individual patient data meta-analysis of 9153 patients into French by author J. Robert**

Impact des traitements de la tuberculose pulmonaire multirésistante sur le devenir des patients : une méta-analyse sur les données individuelles de 9153 patients

Groupe collaboratif pour la méta-analyse sur données individuelles de la tuberculose MDR

Résumé

Contexte. Le traitement de la tuberculose multirésistante (TB MDR) est long, cher, associé à des effets secondaires et a généralement un faible taux de succès. Nous avons conduit une méta-analyse sur données individuelles afin d'évaluer l'impact du nombre et du type d'antibiotiques, ainsi que de la durée du traitement sur le devenir des cas de TB MDR.

Méthode. Trois revues systématiques récentes ont été utilisées pour identifier les études qui rapportaient le devenir de cas de TB MDR confirmés par la microbiologie. Les auteurs de ces études ont été contactés afin de recueillir les données individuelles de chaque cas, dont les caractéristiques cliniques, les traitements reçus et le devenir. Un modèle de méta-régression logistique à effets aléatoires a été utilisé pour évaluer la probabilité ajustée (odds ratio ajusté, ORa) de succès thérapeutique.

Résultats. Des données sur le traitement et le devenir des malades ont été fournies pour 9153 cas de TB MDR provenant de 32 études observationnelles. Par rapport à l'échec ou la rechute, le succès thérapeutique était associé à l'utilisation des fluoroquinolones de dernière génération (ORa: 2,5 [intervalle de confiance à 95%: 1,1-6,0]), d'ofloxacine (ORa: 2,5 [1,6-3,9]), d'éthionamide ou protionamide (ORa: 1,7 [1,3-2,3]), d'au moins quatre antibiotiques potentiellement actifs dans la phase intensive initiale (ORa: 2,3 [1,3-3,9]) et d'au moins trois antibiotiques potentiellement actifs dans la phase d'entretien (ORa: 2,7 [1,7-4,1]). Des résultats similaires étaient obtenus lorsque le succès thérapeutique était comparé à un échec, une rechute ou le décès du malade, le succès étant associé à l'utilisation de fluoroquinolones de dernière génération (ORa: 2,7 [1,7-4,3]), d'ofloxacine (ORa: 2,3 [1,3-3,8]), d'éthionamide ou de protionamide (ORa: 1,7 [1,4-2,1]), d'au moins quatre antibiotiques potentiellement actifs dans la phase intensive initiale (ORa: 2,7 [1,9-3,9]) et d'au moins trois antibiotiques potentiellement actifs dans la phase d'entretien (ORa: 4,5 [3,4-6,0]).

Conclusion. Dans cette méta-analyse sur données individuelles provenant d'études observationnelles, l'utilisation de certaines fluoroquinolones, d'éthionamide ou prothionamide, et un traitement par un grand nombre d'antibiotiques actifs étaient associés à un taux de succès thérapeutique et de survie plus élevés. Néanmoins, ces résultats doivent être rapidement confirmés par des essais randomisés afin d'optimiser le traitement des TB MDR.
