# Supplementary material for: Multidrug Resistant Pulmonary Tuberculosis Treatment Regimens and Patient Outcomes: An Individual Patient Data Meta-analysis of 9,153 Patients
Source: PLoS Med. 2012 Aug 28;9(8):e1001300. doi: 10.1371/journal.pmed.1001300 (PMC3429397; doi:10.1371/journal.pmed.1001300)
Supplement: Alternative Language Abstract S3 — German translation of the abstract by CL. (DOCX) [file pmed.1001300.s003.docx]

**Alternative Language Abstract 3: German**

**Translation of the abstract Multidrug-resistant pulmonary tuberculosis treatment regimens and patient outcomes: an individual patient data meta-analysis of 9153 patients into German by author Christoph Lange**

Behandlungserfolg der multiresistenten Tuberkulose (MDR-TB) in Abhängigkeit der verwendeten Therapie:

Eine Meta Analyse an individuellen Daten von 9152 Patienten.

Zusammenfassung:

Hintergrund: Die Behandlung von Patienten mit multiresistenter Tuberkulose (MDR-TB) ist langwierig, nebenwirkungsreich, kostspielig und ist generell nur wenig erfolgreich. Wir haben eine Meta-Analyse an individuellen Patientendaten durchgeführt, um die Bedeutung der Wahl verschiedener Medikamente, deren Anzahl und die Dauer der Therapie für den Erfolg der Behandlung der MDR-TB zu ermitteln.

Methoden: Auf der Basis von drei kürzlich veröffentlichten systematischen Reviews wurden Studien mit mikrobiologisch bestätigten Fällen einer MDR-TB ermittelt, in denen der Ausgang der Behandlung dokumentiert wurde. Die Autoren dieser Studien wurden kontaktiert um individuelle Patientendaten, inklusive klinischer Angaben, Angaben zur Therapie und zum Ausgang der Behandlung zusammenzuführen. Um die adjustierte Wahrscheinlichkeit des Therapieerfolges abzuschätzen, wurde die Methode der Random-effects multivariaten logistischen Meta-Regression angewendet.

Ergebnisse: Für 9153 Patienten mit einer MDR-TB wurden aus 32 Observationsstudien adäquate Daten zur Therapie und zum Ausgang der Behandlung übermittelt. Ein Behandlungserfolg, im Vergleich zum Therapieversagen/Rezidiv, war mit den folgenden Variablen verbunden: Fluroquinolone der späteren Generation (adjustiertes Quotenverhältnis – engl. adjusted Odds Ratio (aOR)- 2.5 [95% Konfidenzinterval: 1.1, 6.0]), Ofloxacin (aOR: 2.5 [1.6, 3.9]), Ethionamid oder Prothionamid (aOR: 1.7 [1.3, 2.3]), vier oder mehr wahrscheinlich wirksamen Medikamenten in der initialen Phase der Therapie (aOR: 2.3 [1.3, 3.9]), und drei oder mehr wahrscheinlich wirksamen Medikamenten in der Erhaltungsphase der Therapie (aOR: 2.7 [1.7, 4.1]). Ähnliche Ergebnisse wurden für den Vergleich eines Behandlungserfolgs zu einem Therapieversagen/Rezidiv oder zum Tod beobachtet:

Fluroquinolone der späteren Generation (aOR: 2.7 [1.7, 4.3]), Ofloxacin (aOR: 2.3 [1.3, 3.8]), Ethionamid oder Prothionamid (aOR: 1.7 [1.4, 2.1]), vier oder mehr wahrscheinlich wirksamen Medikamenten in der initialen Phase der Therapie (aOR: 2.7 [1.9, 3.9]), und drei oder mehr wahrscheinlich wirksamen Medikamenten in der Erhaltungsphase der Therapie (aOR: 4.5 [3.4, 6.0]).

Schlussfolgerung: In dieser Meta-Analyse basierend auf individuellen observierten Patientendaten waren Behandlungserfolg und Überleben mit der Verwendung bestimmter Fluroquinolone, Ethionamid oder Protionamid und einer größeren Anzahl wirksamer Medikamente in der Therapie assoziiert. Randomisierte Studien werden dringend benötigt um die Therapie der MDR-TB zu optimieren.
