# Supplementary material for: Multidrug Resistant Pulmonary Tuberculosis Treatment Regimens and Patient Outcomes: An Individual Patient Data Meta-analysis of 9,153 Patients
Source: PLoS Med. 2012 Aug 28;9(8):e1001300. doi: 10.1371/journal.pmed.1001300 (PMC3429397; doi:10.1371/journal.pmed.1001300)
Supplement: Alternative Language Abstract S4 — Italian translation of the abstract by GS and GBM. (DOCX) [file pmed.1001300.s004.docx]

**Alternative Language Abstract 4:Italian**

**Translation of the abstract Multidrug-resistant pulmonary tuberculosis treatment regimens and patient outcomes: an individual patient data meta-analysis of 9153 patients into Italian by author Giovanni Sotgiu, Giovanni Migliori**

Regimi terapeutici ed outcome clinici nella tubercolosi multi-farmaco resistente:

Meta-analisi di dati individuali su 9153 pazienti.

Abstract:

Background: Il trattamento della tubercolosi multi-farmaco resistente (MDR-TB) è di lunga durata, caratterizzato da frequenti eventi avversi, costoso, e non modifica nella totalità dei casi la prognosi sfavorevole della malattia. Abbiamo eseguito una meta-analisi di dati individuali allo scopo di valutare l’impatto del tipo, numero e durata dei farmaci anti-tubercolari sull’esito di trattamento della MDR-TB.

Metodi: Per identificare gli studi che riportavano gli outcome del trattamento in casi di MDR-TB confermati batteriologicamente, sono state utlizzate tre recenti revisioni sistematiche. Gli autori dello studio sono stati contattati individualmente per sollecitare l’invio dei dati dei pazienti, che includevano tipo ed outcome del trattamento, ed altre caratteristiche individuali. Una meta-regressione logistica multi-variata ad effetti casuali è stata utilizzata per stimare gli odds di successo terapeutico aggiustati per variabili confondenti.

Risultati: Dati adeguati circa tipologia di trattamento ed outcomes sono stati ottenuti su 9153 pazienti con MDR-TB arruolati in 32 studi osservazionali. Il successo terapeutico rapportato al fallimento/ricaduta clinica è risultato essere associato ad uso di fluorochinolonici di ultima generazione (Odds Ratio aggiustato (aOR): 2.5 [intervallo di confidenza al 95%: 1.1, 6.0]), ofloxacina (aOR: 2.5 [1.6, 3.9]), etionamide o protionamide (aOR: 1.7 [1.3, 2.3]), uso di 4 o più farmaci efficaci nella fase intensiva (aOR: 2.3 [1.3, 3.9]), ed uso di 3 o più farmaci efficaci nella fase di continuazione (aOR: 2.7 [1.7, 4.1]). Analoghi risultati sono stati ottenuti per l’associazione di successo terapeutico rapportato a fallimento/ricaduta clinica o morte: chinolonici di ultima generazione, (aOR: 2.7 [1.7, 4.3]), ofloxacina (aOR: 2.3 [1.3, 3.8]), etionamide o protionamide (aOR: 1.7 [1.4, 2.1]), uso di 4 o più farmaci efficaci nella fase intensiva (aOR: 2.7 [1.9, 3.9]), e uso di 3 o più farmaci efficaci nella fase di continuazione (aOR: 4.5 [3.4, 6.0]).

Conclusioni: In questa meta-analisi di dati individuali, basata su studi osservazionali, soppravvivenza e migliorato successo terapeutico nelle forme di MDR-TB sono risultate essere associate all’uso di fluorochinolonici, etionamide o protionamide e ad un numero elevato di farmaci efficaci. Tuttavia sono necessari studi randomizzati in tempi brevi allo scopo di ottimizzare il trattamento della MDR-TB.
