# Supplementary material for: Multidrug Resistant Pulmonary Tuberculosis Treatment Regimens and Patient Outcomes: An Individual Patient Data Meta-analysis of 9,153 Patients
Source: PLoS Med. 2012 Aug 28;9(8):e1001300. doi: 10.1371/journal.pmed.1001300 (PMC3429397; doi:10.1371/journal.pmed.1001300)
Supplement: Alternative Language Abstract S5 — Korean translation of the abstract by T-SS, W-JK, and J-JY. (DOCX) [file pmed.1001300.s005.docx]

**Alternative Language Abstract 5: Korean**

**Translation of the abstract “Multidrug-resistant pulmonary tuberculosis treatment regimens and patient outcomes: an individual patient data meta-analysis of 9153 patients.” Into Korean by authors Tae-Sun Shim, Won-Jung Koh, Jae-Joon Yim**

다제내성 폐결핵 치료 약제에 따른 치료 결과: 9153명의 환자 개인별 자료 메타분석

배경: 다제내성결핵의 치료는 오랜 기간이 필요하고, 부작용이 흔하고, 비용이 많이 소요되며, 일반적으로 치료결과가 불량하다. 이 연구에서 환자 개인별 자료를 이용한 메타분석을 통해 치료 약제의 종류, 갯수 및 사용 기간이 치료 결과에 미치는 영향을 분석했다.

방법: 최근 출판된 3편의 체계적 고찰 문헌을 이용하여 미생물학적으로 확진된 다제내성결핵환자의 치료 결과를 보고한 논문을 확인하였다. 각 논문의 저자와 접촉하여 환자 개인별 임상적 특성, 치료 약제 및 치료 결과를 확인하였다. 치료 성공에 관한 보정된 교차비를 adjusted OR (aOR) 구하기 위해 랜덤 효과 다변수 로지스틱 메타 회귀분석을 시행했다.

결과: 32개의 관찰연구에 포함된 다제내성결핵환자 9153명의 치료 방법과 결과에 관한 자료를 수집했다. 치료 실패/재발과 비교하였을 때 치료 성공은 최근 개발된fluoroquinolone (aOR:2.5 [95% 신뢰구간: 1.1, 6.0])의 사용, ofloxacin (aOR: 2.5 [1.6, 3.9])의 사용, ethionamide 혹은 prothionamide (aOR: 1.7 [1.3, 2.3])의 사용, 초기집중치료기에 효과적일 것으로 생각되는 약제 4개 이상의 사용 (aOR: 2.3 [1.3, 3.9]), 유지치료기에 효과적일 것으로 생각되는 약제 3개 이상의 사용과(aOR: 2.7 [1.7, 4.1]) 연관되어 있었다. 치료실패/재발 및 사망과 비교하였을 때도 치료 성공과 관련된 변수는 비슷했는데, 최근 개발된fluoroquinolone의 사용(aOR: 2.7 [1.7, 4.3]), ofloxacin(aOR: 2.3 [1.3, 3.8]), ethionamide 혹은 prothionamide의 사용(aOR: 1.7 [1.4, 2.1]), 초기집중치료기에 효과적일 것으로 생각되는 약제 4개 이상의 사용(aOR: 2.7 [1.9, 3.9]), 유지치료기에 효과적일 것으로 생각되는 약제 3개 이상의 사용이(aOR: 4.5 [3.4, 6.0]) 치료 성공과 연관되어 있었다.

결론: 관찰 연구에 포함된 환자 개인별 자료 메타분석을 통해 다제내성결핵 치료 성공률과 생존율의 향상은 특정 fluoroquinolone의 사용, ethionamide 혹은 prothionamide의 사용, 많은 유효 약제 사용 갯수와 연관됨을 확인하였다. 그러나 다제내성결핵 치료의 최적화를 위해서는 무작위배정 연구가 시급하다.
