# Supplementary material for: Multidrug Resistant Pulmonary Tuberculosis Treatment Regimens and Patient Outcomes: An Individual Patient Data Meta-analysis of 9,153 Patients
Source: PLoS Med. 2012 Aug 28;9(8):e1001300. doi: 10.1371/journal.pmed.1001300 (PMC3429397; doi:10.1371/journal.pmed.1001300)
Supplement: Alternative Language Abstract S6 — Spanish translation of the abstract by DP, MLGG, JS-O, AP-d-L, MHV, and CP-G. (DOCX) [file pmed.1001300.s006.docx]

**Alternative Language Abstract 6: Spanish**

**Translation of the abstract “Multidrug-resistant pulmonary tuberculosis treatment regimens and patient outcomes: an individual patient data meta-analysis of 9153 patients.” Into Spanish by authors Domingo Palmero, Lourdes García-García, Jose Sifuentes-Osornio, Alfredo Ponce de Leon, Mario H Vargas and Carlos Pèrez-Guzmán.**

Regímenes de tratamiento para la tuberculosis pulmonar multidrogorresistente y respuesta clínica: metaanálisis de datos individuales de 9153 pacientes

“Grupo Colaborativo para el Metaanálisis de Datos Individuales de Pacientes con TBMDR”

Miembros (en orden alfabético):

Resumen:

Antecedentes: El tratamiento de la tuberculosis multidrogorresistente (TBMDR) es prolongado, tóxico, costoso y en general tiene pobres resultados. Realizamos un metaanálisis de datos individuales de pacientes para evaluar el impacto que tienen el tipo, número y tiempo de uso de los fármacos utilizados para tratar la TBMDR sobre los resultados del tratamiento.

Métodos: Se utilizaron tres revisiones sistemáticas recientes para identificar estudios que reportaran resultados del tratamiento de TBMDR confirmada microbiológicamente. Los autores de los estudios fueron contactados para solicitar los datos individuales de los pacientes, incluyendo características clínicas, tratamientos administrados y desenlaces. A través de metarregresión logística multivariada se estimaron los momios ajustados de éxito del tratamiento.

Resultados: Se obtuvieron datos adecuados acerca del tratamiento y resultado de 9153 pacientes con TBMDR provenientes de 32 estudios observacionales. El éxito del tratamiento, comparado con fracaso/recaída, estuvo asociado con el uso de: quinolonas de generaciones recientes [razón de momios ajustada (aOR): 2.5 (intervalo de confianza al 95%: 1.1, 6.0)], ofloxacina [aOR: 2.5 (1.6, 3.9)], etionamida o protionamida [aOR: 1.7 (1.3, 2.3)], cuatro o más drogas posiblemente eficaces en la fase intensiva inicial [aOR: 2.3 (1.3, 3.9)] y tres o más drogas posiblemente eficaces en la fase de continuación [aOR: 2.7 (1.7, 4.1)]. Resultados similares fueron observados para la asociación de éxito del tratamiento comparado con fracaso/recaída o muerte: quinolonas de generaciones recientes [aOR: 2.7 (1.7, 4.3)], ofloxacina [aOR: 2.3 (1.3, 3.8)], etionamida o protionamida [aOR: 1.7 (1.4, 2.1)], cuatro o más drogas posiblemente eficaces en la fase intensiva inicial [aOR: 2.7 (1.9, 3.9)] y tres o más drogas posiblemente eficaces en la fase de continuación [aOR: 4.5 (3.4, 6.0)].

Conclusiones: En este metaanálisis de datos individuales de pacientes a partir de estudios observacionales se encontró que el uso de ciertas fluoroquinolonas, de etionamida o protionamida y de un mayor número total de fármacos eficaces se asoció con una mayor probabilidad de éxito del tratamiento y supervivencia en pacientes con TBMDR. Sin embargo, se necesitan urgentemente estudios clínicos aleatorizados para optimizar el tratamiento de la TBMDR.
