# Supplementary material for: Multidrug Resistant Pulmonary Tuberculosis Treatment Regimens and Patient Outcomes: An Individual Patient Data Meta-analysis of 9,153 Patients
Source: PLoS Med. 2012 Aug 28;9(8):e1001300. doi: 10.1371/journal.pmed.1001300 (PMC3429397; doi:10.1371/journal.pmed.1001300)
Supplement: Alternative Language Abstract S7 — Japanese translation of the abstract by MN and YS. (DOCX) [file pmed.1001300.s007.docx]

**Alternative Language Abstract 7: Japanese**

**Translation of the abstract “Multidrug-resistant pulmonary tuberculosis treatment regimens and patient outcomes: an individual patient data meta-analysis of 9153 patients.” Into Japanese by authors Masa Narita and Yuji Shiraishi**

要約

背景：多剤耐性結核の治療は、長期にわたり，副作用も多く，高額になり、かつ、治療結果が一般的に悪いことが多い。この、メタアナリシスでは、個々の患者のデータを調べ、多剤耐性結核の治療に使われた薬剤のタイプ、数、そして、期間が治療成績に与えた影響を調べた。

方法：まず、最近の３つのシステマチィックレビューを使い、微生物検査で確認された多剤耐性結核を扱った論文を選び出した。その論文の著者たちに連絡をとし、臨床的特長、施された治療、治療結果等の個々の患者のデータを集積した。変量多重ロジスチィック回帰分析を使い、治療成功の調整オッズ比を計算した。

結果：３２の論文より、９１５３人の患者の治療および結果のデータが集められた。治療成功例は、治療不成功例・再発例に比べ、以下のものの使用が関係していた　：後世代のニューキノロン　（調整オッズ比: 2.5 [95%信頼区間: 1.1, 6.0]), 　オフロキサシン　(調整オッズ比: 2.5 [1.6, 3.9]), 　エチオナミドあるいは プロエチオナミド (1.7 [1.3, 2.3]),　治療初期に４剤以上の有効な薬剤を使うこと(2.3 [1.3, 3.9])、治療維持期に３剤以上の有効な薬剤を使うこと(2.7 [1.7, 4.1])。同様な結果が、治療成功例を、治療不成功例・再発例および死亡例に比べた時にも見られた。後世代のニューキノロン　（調整オッズ比: 2.7 [1.7, 4.3]), オフロキサシン (2.3 [1.3, 3.8]), エチオナミドあるいは プロチオナミド (1.7 [1.4, 2.1]),　治療初期に４剤以上の有効な薬剤を使うこと(2.7 [1.9, 3.9]),、治療維持期に３剤以上の有効な薬剤を使うこと(4.5 [3.4, 6.0]).

結論：この観察的研究に基づく個々の患者のデータを使ったメタアナリシスによると、ある種のニューキノロン、エチオナマイドあるいは プロチオナマイド、そして、より数の多い有効な薬剤を使用をすることによることが、多剤耐性結核患者の治療結果の改善に関係ある。しかしながら、多剤耐性結核の治療最適化には、ランダム化比較臨床試験が、早急に必要とされる。
