# Supplementary material for: Multidrug Resistant Pulmonary Tuberculosis Treatment Regimens and Patient Outcomes: An Individual Patient Data Meta-analysis of 9,153 Patients
Source: PLoS Med. 2012 Aug 28;9(8):e1001300. doi: 10.1371/journal.pmed.1001300 (PMC3429397; doi:10.1371/journal.pmed.1001300)
Supplement: Alternative Language Abstract S8 — Russian translation of the abstract by DM. (DOC) [file pmed.1001300.s008.doc]

**Alternative Language Abstract 8: Russian**

**Translation of the abstract “Multidrug-resistant pulmonary tuberculosis treatment regimens and patient outcomes: an individual patient data meta-analysis of 9153 patients.” Into Russian by author Salmaan Keshavjee and Irina Gelmanova**

**Влияние схем лечения пациентов, страдающих легочным туберкулезом с множественной лекарственной устойчивостью, на исходы терапии: данные мета-анализа индивидуальных данных 9153 пациентов.**

Ключевые слова: туберкулез, туберкулез с множественной лекарственной устойчивостью, мета-анализ

**Резюме:**

Введение: Лечение туберкулеза с множественной лекарственной устойчивостью (МЛУ-ТБ) является дорогим, длительным, ассоциируется с большим количеством побочных эффектов и зачастую заканчивается неблагоприятным исходом. Мы провели мета-анализ, основанный на индивидуальных данных пациентов, для оценки влияния дозировок и длительности назначения препаратов, применяемых для лечения МЛУ-ТБ, на исходы лечения.

Методы: Три опубликованных в последнее время систематических обзора были использованы для поиска исследований, описывающих результаты лечения микробиологически подтвержденного МЛУ-ТБ. В дальнейшем от авторов исследований были запрошены индивидуальные данные пациентов, включая клинические характеристики, схемы лечения, и результаты. Для оценки скорректированного отношения шансов успешного лечения (сОШ) использовалась модель случайных эффектов мультифакторной логистической мета- регрессии.

Результаты: Необходимые данные по лечению и исходам были предоставлены по 9153 пациентам с МЛУ-ТБ из 32 обсервационных исследований. Более высокий показатель соотношения успешного лечения к неудачам / рецидивам, был связан с использованием: последних поколений фторхинолонов (сОШ): 2,5 [95% доверительный интервал: 1,1; 6,0]), офлоксацина (cОШ: 2,5 [1,6; 3,9] ), этионамида или протионамида (cОШ: 1,7 [1,3; 2,3]), использованием четырех или более эффективных лекарственных препаратов в интенсивной фазе (cОШ: 2,3 [1,3; 3,9]), и трех или более эффективных лекарственных препаратов в фазе продолжения (cОШ: 2,7 [1,7; 4,1]). Аналогичные ассоциации были отмечены и для показателя отношения излеченных к неудачам/рецидивам и смертельным исходам: использование фторхинолонов последнего поколения (сОШ: 2,7 [1,7; 4,3]), офлоксацина (cОШ: 2,3 [1,3; 3,8]), этионамида или протионамида (сОШ : 1,7 [1,4; 2,1]), использование четырех или более эффективных лекарственных препаратов в интенсивную фазу (сОШ: 2,7 [1,9; 3,9]), и трех или более эффективных лекарственных препаратов в фазу продолжения лечения (сОШ: 4,5 [3,4; 6,0]).

Выводы: При проведении мета-анализа индивидуальных данных пациентов из обсервационных исследований более высокий показатель успешного лечения и выживания ассоциировался с применением определенных фторхинолонов, этионамида или протионамида, и большего количества эффективных противотуберкулезных препаратов в схеме лечения. Тем не менее, для создания оптимальных схем МЛУ терапии имеется срочная потребность в проведении рандомизированных исследований.
