# Supplementary material for: Multidrug Resistant Pulmonary Tuberculosis Treatment Regimens and Patient Outcomes: An Individual Patient Data Meta-analysis of 9,153 Patients
Source: PLoS Med. 2012 Aug 28;9(8):e1001300. doi: 10.1371/journal.pmed.1001300 (PMC3429397; doi:10.1371/journal.pmed.1001300)
Supplement: Table S1 — (a) Overview of settings of 32 studies included in individual patient data meta-analysis of MDR-TB supplemental tables (for on-line supplement—references for included studies are found in main text. (b) Overview of 35 studies excluded from individual patient data meta-analysis of MDR-TB. (DOC) [file pmed.1001300.s009.doc]

**Supplemental Tables (for on-line supplement – references for included studies are found in main text.**

**Supplemental Table 1a. Overview of settings of 32 studies Included in Individual Patient Data Meta-analysis of MDR-TB**

| **First Author**  **(Contact Person)**  **Reference** | **Years of Study*** | **Location** | **Catchment Area** | **Source of MDR-TB cases** | **Type of Drug Regimen**  **(with second line drugs unless marked)** | **Number of subjects with MDR-TB eligible for present meta-analysis**** |
| --- | --- | --- | --- | --- | --- | --- |
| *Avendaño (Avendaño) [16]* | 2000-2009 | Canada  (Toronto) | Hospital | Referral centre | Individualized | 72 |
| *Burgos (Burgos)[17]* | 1983-2000 | USA  (San Francisco) | City | TB Section of San Francisco Department of Public Health | Individualized | 45 |
| *Chan (Strand)[18,19]* | 1984-1998 | USA  (Colorado) | Hospital | National Jewish Medical and Research Center | Individualized | 194 |
| *Chiang (Enarson)[20]* | 1992-1996 | Taiwan  (Taipei) | City  (network) | *Mycobacteriology* Laboratory of the Chronic Disease Control Bureau (linked with network of public health nurses in townships & villages) | Individualized | 125 |
| Cox (Cox) [21] | 2003-2005 | Uzbekistan | Community  (multi-center) | Nukus City hospital and outpatient clinics and DOTS clinics in Chimbay district | Individualized | 77 |
| *De Riemer (Garcia-Garcia)[22]* | 1994-2009 | Mexico  (Veracruz) | Community  (multi-center) | National TB Program | Standardized  (43 patients received first-line drugs only) | 47 |
| *Escudero (Pena) [23]* | 1998-2000 | Spain  (Madrid) | Hospital | University Hospital | Individualized | 18 |
| *Geerligs (van der Werf) [24]* | 1987-1988, 1998-2008 | The Netherlands | Community  (multi-center) | Two specialized referral centers for TB | Individualized | 43 |
| *Granich/ Banerjee (Flood) [25,26]* | 1994-2006 (paper goes to 2003) | USA  (California) | State (California) | State TB Program data | Individualized | 100 |
| *Holtz (Van der Walt) [27]* | 2000-2004 | South Africa  (All centres) | Country  (multi-center) | National TB Program | Standardized | 2174 |
| *DH Kim (Shim) [28]* | 2000-2002 | South Korea  (Seoul) | Country  (multi-center) | National TB Hospitals, Korean National TB Association Chest Clinics & eight randomly selected university hospitals | Individualized | 1288 |
| *HR Kim (Yim) [29]* | 1980-2007 | South Korea  (Seoul) | Hospital | University-affiliated tertiary care referral hospital (Seoul National University Hospital) | Individualized | 182 |
| *Kwon (Koh) [30]* | 1995-2005 | South Korea  (Seoul) | Hospital | University-affiliated Tertiary Care Hospital (Samsung Medical Center) | Individualized | 129 |
| *Leimane/Holtz/Riekstina [31–33]* | 2000-2004 | Latvia  (Stopinu Novads) | Clinic | National TB Control Program | Individualized | 945 |
| *Lockman (Viiklepp) [34]* | 2000-2002 | Estonia  (All centres) | Country | National TB Program | Individualized | 218 |
| *Masjedi (Tabarsi) [35]* | 2002-2006 | Iran | Country  (multi-center) | National Mycobacteriological Reference Laboratory | Standardized | 27 |
| *Migliori (Centis) [36,37]* | 2001-2004 | Italy | Country  (multi-centre) | TB Clinical Reference Centers in Italy | Individualized | 83 |
| *Mitnick (Mitnick) [38,39]* | 1996-2002 | Peru  (Lima) | City  (multi-centre) | Peruvian National TB Programme | Individualized | 654 |
| *Munsiff/Li (Ahuja)[40,41]* | 1992-1997 | USA  (New York) | City and State | Local and State TB Program | Individualized | 671 |
| *Narita (Narita)[42]* | 1993-1997 | USA  (Florida) | State | Florida State TB Laboratory | Individualized | 66 |
| *O’Riordan (Pasvol)[43]* | 1982-2004 | UK  ( London) | Hospital | Northwick Park Hospital (local population, Health Care Unit at London’s Heathrow & Gatwick Airport, and tertiary referrals from other hospitals) | Individualized | 28 |
| *Palmero (Palmero)[44]* | 1996-1999 | Argentina  (Buenos Aires) | Hospital | National Reference Hospital for Infectious Diseases (Hospital Muñiz) | Individualized | 112 |
| *Park (Seung)[45]* | 1998-2002 | South Korea  (Masan) | Hospital | National TB Hospital (National Masan Tuberculosis Hospital) | Standardized | 131 |
| *Perez-Guzman (Vargas) [46]* | 1994-1995 | Mexico | Hospital | Pulmonary TB Clinics of the Instituto Nacional de Enfermedades Respiratorias | Individualized | 33 |
| *Quy (Dang/Cobelens) [47]* | 1998-2000 | Vietnam  (Ho Chi Minh City) | City  (multi-center) | National TB Control Program | Standardized  (First-line drugs only) | 157 |
| *Schaaf (Schaaf) [48]* | 1998-2002 | South Africa  (Western Cape, Capetown Metropole, West Coast) | Multi-regional  (multi-center) | MDR-TB clinics &  Local hospitals | Individualized | 36 |
| *Shin (Shin)[49]* | 2000-2004 | Russian Federation (Tomsk) | Oblast  (multi-center) | Tomsk Oblast TB Services, Tomsk Penitentiary Services and Tomsk TB Hospital | Individualized | 535 |
| *Shiraishi (Shiraishi) [50]* | 2000-2007 | Japan  (Tokyo) | Hospital | Fukujuji Hospital | Individualized | 61 |
| *Tupasi (Quelapio) [51,52]* | 1999-2003 | Philippines | Clinic | Makati Medical Center Dots Clinic | Individualized | 159 |
| *Uffredi (Robert) [53]* | 1998-1999 | France  (Paris) | Multi-regional | National Reference  Center | Individualized | 41 |
| *Van Deun (Aung Maug) [54,55]* | 1997-2010 | Bangladesh | Country  (multi-center) | Bangladesh National TB Program (& Damien Foundation) | Standardized | 603 |
| *Yew (Leung)[56,57]* | 1990-1997 | Hong Kong | Hospital | Tertiary Referral Hospital for TB (Grantham Hospital) | Individualized | 99 |

*Defined by start date of MDR-TB treatment.

** The data sets initially included 25 patients or more with MDR TB. After excluding patients with extra pulmonary disease, XDR, or who were missing information about treatment or outcomes there were fewer patients from each study – in some cases less than 25.

**Supplemental Table 1b. Overview of 35 studies excluded from Individual Patient Data Meta-analysis of MDR-TB**

**For excluded studies (Table S1b), references are included here)**

| **First author** | **Publication year** | **Year(s) of treatment and follow-up** | **Country** | **Patients (N)** | **Cured (%)** | **Notes** |
| --- | --- | --- | --- | --- | --- | --- |
| Bartu,V | 2007 | 2001-2004 | Czech Republic | 45 | 53% |  |
| Bashar, M | 2001 | 1987-1997 | USA - NYC | 28 | NA | Outcomes aggregated for MDR TB and TB patients |
| Bloch, AB | 1999 | 1993 | USA | 447 | 36% |  |
| Bonnet, M | 2005 | 1997-2002 | Russia, Georgia, Azerbaijan, Uzbekistan, Turkmenistan, Kazakhstan | 282 | 71% | Outcomes of MDR TB and all TB patients aggregated except at Kemerovo and Abkhasia sites |
| Coninx, R | 1999 | 1995-1998 | Azerbaijan | 60 | 25% |  |
| Corlan, E | 1997 | NA | Romania | 32 | NA | Outcomes aggregated for MDR and mono-resistant TB |
| Drobniewski, F | 2002 | 1996-1997 | UK | 90 | NA |  |
| Eker, B | 2008 | 2004-2006 | Germany | 177 | 59% |  |
| Espinal, MA | 2000 | 1994-1996 | Dominican Republic, Hong Kong, Italy, Russia, South Korea, Peru | 353 | 41% |  |
| Fischl, MA | 1992 | 1988-1990 | USA | 40 | NA | No outcomes. All patients were HIV+ |
| Flament-Saillour, M | 1999 | 1994-1996 | France | 40 | 42% |  |
| Gandhi, NR | 2006 | 2005-2006 | South Africa | 168 | NA | No Outcomes. Surveillance study |
| Goble, M | 1993 | 1973-1983 | USA | 119 | NA | No outcomes |
| Hersi, A | 1999 | 1989-1998 | Canada | 24 | 33% |  |
| Kim, HJ | 2001 | 1988-1996 | South Korea | 1011 | 48% |  |
| Kritski, AL | 1997 | 1986-1990 | Brazil | 77 | 27% | Cured = remained disease-free after mean 20.5 months |
| Lan, NTN | 2001 | 1991-1994 | Vietnam | 42 | 14 (33%) |  |
| Lee, CN | 1996 | 1987-1989 | Taiwan | 36 | 17 (47%) |  |
| Mangunnegoro, H | 1999 | 1995-1996 | Indonesia | 58 | 39 (67%) |  |
| Migliori, GB | 2002 | 1995-1999 | Russia | 76 | 18 (24%) |  |
| Nathanson, E | 2006 | 1999-2001 | Estonia, Latvia, Peru, Philippines, Russia | 1047 | 70% |  |
| Olle-Goig, JE | 2005 | 1983-1993 | Bolivia | 143 | 29% |  |
| Park, MM | 1996 | 1983-1994 | USA - NYC | 173 | 56% | Cured = number alive who completed treatment |
| Pathan, AJ | 1996 | NA | Pakistan | 23 | 18 (78%) |  |
| Prete, S | 1992 | 1986-1989 | France | 23 | 14 (61%) |  |
| Sosna, J | 1999 | 1985-1994 | Israel | 57 | 21 (37%) |  |
| Sung, SW | 1999 | 1994-1998 | South Korea | 27 | 26 (92%) | all patients also underwent pulmonary resection |
| Surucuoglu, S | 2005 | 1997-2003 | Turkey | 26 | 14 (54%) |  |
| Tahalogu, K | 2001 | 1992-1999 | Turkey | 158 | 121 (77%) |  |
| Takeda, S | 2005 | 1988-2003 | Japan | 26 | 23 (89%) | all patients also underwent pulmonary resection |
| Telzak, EE | 1995 | 1991-1995 | USA - NYC | 25 | 16 (64%) |  |
| Törün, T | 2005 | 1992-2004 | Turkey | 263 | 204 (78%) |  |
| Turett, GS | 1995 | 1991-1993 | USA - NYC | 38 | 24 (63%) | Cured = number with microbiological and clinical response to treatment |
| Van Leuven, M | 1997 | 1990-1995 | South Africa | 62 | 4 (7%) | all patients also underwent pulmonary resection |
| Ward, HA | 2005 | 1989-2000 | Vietnam | 44 | 38 (86%) |  |
| Total (N) |  |  |  | **5340** |  |  |

**References for Table S1b (Excluded Studies)**

(1) Bartu V. Multidrug-resistant tuberculosis in the Czech Republic: strategy and therapeutic outcomes. Eur J Clin Microbiol Infect Dis 2007;26:603-5.

(2) Bashar M, Alcabes P, Rom WN, Condos R. Increased incidence of multidrug-resistant tuberculosis in diabetic patients on the Bellvue Chest Service, 1987 to 1997. Chest 2001;120(5):1514-9.

(3) Bloch AB, Cauthen GM, Simone PM, Kelly GD, Dansbury KG, Castro KG. Completion of tuberculosis therapy for patients reported in the United States in 1993. Int J Tuberc Lung Dis 1999;3(4):273-80.

(4) Bonnet M, Sizaire V, Kebede Y, Janin A, Doshtetov D, Mirzoian B, Arzumanian A, Muminov T, Iona E, Rigouts L, Rusch-Gerdes S, Varaine F. Does one size fit all? Drug resistance and standard treatments: results of six tuberculosis programmes in former Soviet countries. Int J Tuberc Lung Dis 2005;9(10):1147-54.

(5) Coninx R, Mathieu C, Debacker M, Mirzoev F, Ismaelov A, de Haller R, Meddings DR. First-line tuberculosis therapy and drug-resistant *Mycobacterium tuberculosis* in prisons. The Lancet 1999;353:969-73.

(6) Corlan E, Marica C, Macavei C, Stanford JL, Standford CA. Immunotherapy with *Mycobacterium vaccae* in the treatment of tuberculosis in Romania. 2. chronic or relaped disease. Respiratory Medicine 1997:91:21-9.

(7) Drobniewski F, Elktringham I, Graham C, Magee JG, Smith EG, Watt B. A national study of clinical and laboratory factors affecting the survival of patients with multiple drug resistant tuberculosis in the UK. Thorax 2002;57:810-6.

(8) Ecker Barbara, Ortman J, Migliori GB, Sotgiu G, Muetterlein R, Centis R, Hoffmann H, Kirsten D, Schaberg T, Ruesch-Gerdes S, Lange C, for the German TBNET Group. Multidrug- and extensively drug-resistant tuberculosis, Germany. Emerging Infectious Diseases 2008;14(11):1700-6.

(9) Espinal MA, Kim SJ, Suarez PG. Standard short-course chemotherapy for drug-resistant tuberculosis: treatment outcomes in 6 countries. JAMA 2000;283(19):2537-45.

(10) Fischl MA, Daikos GL, Uttamchandani RB, Poblete RB, Moreno JN, Reyes RR, Boota AM, Thompson LM, Clearly TJ, Oldham SA, Saldana MJ, Lai S. Clinical presentation and outcome of patients with HIV infection and tuberculosis caused by multiple-drug-resistant bacilli. Annals of Internal Medicine 1992;117:184-90.

(11) Flament-saillour M, Robert J, Jarlier V, Grosset J. Outcome of multi-drug-resistant tuberculosis in France a nationwide case-control study. Am J Respir Crit Car Med 1999;160:587-93.

(12) Gandhi NR, Moll A, Sturm AW, Pawinski R, Govender T, Lalloo U, Zeller K, Andrews J, Friedland G. Extensively drug-resistant tuberculosis as a cause of death in patients co-infected with tuberculosis and HIV in a rural area of South Africa. The Lancet 2006;368:1575-80.

(13) Goble M, Iseman MD, Madsen LA, Waite D, Ackerson L, Horsburgh, Jr. R. Treatement of 171 patients with pulmonary tuberculosis resistant to Isoniazid and Rifampin. N Engl J Med 1993; 328(8):527-32.

(14) Hersi A, Elwood K, Cowie R, Kunimoto D, Long R. Multidrug-resistant tuberculosis in Alberta and British Columbia, 1989 to 1998. Can Respir J 199;6(2):155-60.

(15) Kim HJ, Hong YP, Kim SJ, Lew WJ, Lee EG. Ambulatory treatment of multidrug-resistant pulmonary tuberculosis patients at a chest clinic. Int J Tuberc Lung Dis 2001;5(12):1129-36.

(16) Kritski AL, Rodrigues de Jesus LS, Andrade MK, Werneck-Barroso E, Vieira MAMS, Haffner A, Riley LW. Retreatment tuberculosis cases factors associated with drug resistance and adverse outcomes. Chest 1997;111:1162-7.

(17) Lan NTN, Iademarco MF, Binkin NJ, Tung LB, Quy HT, Co NV. A case series: initial outcome of persons with multidrug-resistant tuberculosis after treatment with the WHO standard retreatment regimen in Ho Chi Minh City, Vietnam. Int J Tuberc Lung Dis 2001;5(6):575-8.

(18) Lee CN, Lin TP, Chang MF, Jimenez MV, Dolfi L, Olliario P. Rifabutin as salvage therapy for cases of chronic multidrug-resistant pulmonary tuberculosis in Taiwan. Journal of Chemotherapy 1996;8(2):137-43.

(19) Mangunnegoro H, Hudoyo A. Efficacy of low-dose Ofloxacin in the treatment of multidrug-resistant tuberculosis in Indonesia. Chemotherapy 1999;45(suppl 2):19-25.

(20) Migliori GB, Espinal M, Danilova ID, Punga VV, Grzemska M, Raviglione MC. Frequency of recurrence among MDR-TB cases ‘successfully’ treated with standardised short-course chemotherpay. Int J Tuberc Lung Dis 2002;6(10):858-64.

(21) Nathanson E, Weezenbeek CL, Rich ML, Gupta R, Bayona J, Blondal K, Caminero JA, Cegielski JP, Danilovits M, Espinal MA, Hollo V, Jaramillo E, Leimane V, Mitnick CD, Mukherjee JS, Nunn P, Pasechnikov A, Tupasi T, Wells C, Raviglione MC. Multidrug-resistant tuberculosis management in resource-limited settings. Emerging Infectious Diseases 2006;12(9):11389-97.

(22) Ollé-Goig JE, Sandy R. Outcomes of individualised treatment for multidrug-resistant tuberculosis before DOTS-Plus. Int J Tuberc Dis 2005;9(7):765-70.

(23) Park MM, Davis AL, Schluger NW, Cohen H, Rom WN. Outcome of MDR-TB patients, 1983-1993. Am J Respir Crit Care Med 1996;153:317-24.

(24) Pathan AJ, Khan AA. Treatment of short course chemotherapy (SHRZE) failure cases with Kanamycin, Ethionamide and Cycloserine. Pakistan’s J Med Sci 1996;12(2):101-6.

(25) Pretet S, Lebeaut A, Parrot R, Truffort C, Grosset J, Dinh-Xuan AT, G.E.T.I.M (Group for the Study and Treatment of Resistant Mycobacterial Infections). Combined chemotherapy including Rifabutin for Rifampicin and Isoniazid resistant pulmonary tuberculosis. Eur Respir J 1992;5:680-4.

(26) Sonsa J, Schulimzon T, Roznman J, Lidgi M, Lavy A, Ben-Dov IZ, Ben-Dov I. Drug-resistant pulmonary tuberculosis in Israel, a society of immigrants: 1985-1994. Int J Tuberc Lung Dis 3(8):689-94.

(27) Sung SW, Kang CH, Kim YT, Han SK, Shim Y-S, Kim JH. Surgery increased the chance of cure in multi-drug resistant pulmonary tuberculosis. European Journal of Cardio-thoracic Surgery 1999;16:197-93.

(28) Surucuoglu S, Ozkutuk N, Celik P, Gazi H, Dinc G, Kurutepe S, Koroglu G, Havlucu Y, Tuncay G. Drug-resistant pulmonary tuberculosis in western Turkey: prevalence, clinical characterisitcs and treatment outocme. Ann Saudi Med 2005;25(4):313-8.

(29) Tahaoglu K, Torun T, Sevim T, Atac G, Kir A, Karasulu L, Ozmen I, Kapakli N. The treatment of multidrug-resistant tuberculosis in Turkey. N Engl J Med 2001;345(3):170-7.

(30) Takeda S, Maeda H, Hayakawa M, Sawabata N, Maekkura R. Current surgical intervention for pulmonary tuberculosis. Ann Thorac Surg 2005;79:959-63.

(31) Telzak EE, Sepkowitz K, Alpert P, Mannheimer S, Medard F, El-Sadr W, Blum S, Gagliardi A, Salomon N, Turrett G. Multidrug-resistant tuberculosis in patients without HIV infection. N Engl J Med 1995;333(14):907-11.

(32) Torun T, Gungor G, Ozmen I, Bolukbasi Y, Maden E, Bicakci B, Atac G, Sevim T, Tahaoglu K. Side effects associated with the treatment of multidrug-resistant tuberculosis. Int J Tuberc Lung Dis 2005;9(12):1373-7.

(33) Turett GS, Telzak EE, Torian LV, Blum S, Alland D, Weisfuse I, Fazal BA. Improved outcomes for patients with multidrug-resistant tuberculosis. Clinical Infectious Diseases 1995;21:1238-44.

(34) Van Leuven M, De Groot M, Shean KP, Von Oppel UO, Willcox PA. Pulmonary resection as an adjunct in the treatment of multiple drug-resistant tuberculosis. Ann Thorac Surg 1997;63:1368-73.

(35) Ward HA, Marciniuk DD, Hoeppner VH, Jones W. Treatment outcomes of multidrug-resistant tuberculosis among Vietnamese immigrants. Int J Tuberc Lung Dis 2005;9(2):164-9.
