# Supplementary material for: Multidrug Resistant Pulmonary Tuberculosis Treatment Regimens and Patient Outcomes: An Individual Patient Data Meta-analysis of 9,153 Patients
Source: PLoS Med. 2012 Aug 28;9(8):e1001300. doi: 10.1371/journal.pmed.1001300 (PMC3429397; doi:10.1371/journal.pmed.1001300)
Supplement: Table S2 — Dosages of drugs used for MDR-TB treatment at sites of studies included in individual patient data meta-analysis on MDR-TB supplemental tables (for on-line supplement—references for included studies are found in main text. (DOC) [file pmed.1001300.s010.doc]

**Supplemental Tables (for on-line supplement – references for included studies are found in main text**

**Supplemental Table 2. Dosages of drugs used for MDR-TB treatment at sites of studies included in Individual Patient Data Meta-analysis on MDR-TB**

| **First Author (Alt Contact Person)** | **Ethambutol** | **Pyrazinamide** | **Quinolones** | | | **Injectables** | | | | |
| --- | --- | --- | --- | --- | --- | --- | --- | --- | --- | --- |
|  |  |  | **Ofloxacin** | **Levofloxacin** | **Moxifloxacin** | **Amikacin** | **Kanamycin** | | **Streptomycin** | **Capreomycin** |
| *Avendaño (Avendaño) [16]* | 15-25 mg/kg/day | 25 mg/kg/day, max: 2gm/day | Not used | 1000 mg/day | 400 mg/day | 15 mg/kg/day | Not used | | Not used | Not used |
| *Burgos (Burgos)[17]* | 15-25 mg/kg/day | 25 mg/kg/day, max: 2gm/day | 400-800 mg/day | 500 mg/day | Not used | 15 mg/kg/day 5-7 days/week  max 1gm/day | 15 mg/kg/day 5-7 days/week  max 1gm/day | | 15 mg/kg/day 5-7 days/week  max 1gm/day | 15 mg/kg/day 5-7 days/week  max 1gm/day |
| *Chan (Strand)[18,19]* | 25 mg/kg/day for 2 mos, then 15 mg/kg/day | 25-50 mg/kg/day | 600-800 mg/day | 500-750 mg/day | 400 mg/day | 12-15 mg/kg/day  Mon-Fri  22-25 mg/kg/d  if MWF, | Not used | | Not used | Not used |
| *Chiang (Enarson)[20]* | - | - | 300-400 mg, BID | Not used | Not used | Not used | 10-15 mg/kg/day | | 10-15 mg/kg/day | Not used |
| Cox (Cox) [21] | - | 750-2500 mg/day | 600-800 mg/day | Not used | Not used | Not used | - | | - | 500-1000 mg/day |
| *De Riemer (Garcia-Garcia)[22]* | 15-25 mg/kg/day  (max. 1.2 g) | 20-30 mg/kg/day  (max. 1.5-2 g) | Not used | Not used | Not used | Not used | Not used | | 15 mg/kg/day  (max. 1 g) | Not used |
| *Escudero (Pena) [23]* | 15-25 mg/kg/day | 35 mg/kg/day | 800 mg/day | Not used | Not used | 15 mg/kg/day  max 1gm/day | 15 mg/kg/day  max 1gm/day | | 15 mg/kg/day  max 1gm/day | 15 mg/kg/day  max 1gm/day |
| *Geerligs (van der Werf) [24]* | 15-25 mg/kg/day  (max. 1.2 g) | 20-30 mg/kg/day  (max. 1.5-2 g) | Not used | Not used | 400 mg/day | 15 mg/kg/day =750-1,000 mg. After 2004 – 400mg/day | 15 mg/kg/day =750-1,000 mg. After 2004 – 400mg/day | | Not used | Not used |
| *Granich/ Banerjee (Flood) [25,26]* | 15-25 mg/kg/day | 25 mg/kg/day (max 2 grams) | Not used- | 500-1000 mg/day | 400 mg/day | 15 mg/kg/day 5-7 days/week | Not used | | 15 mg/kg/day  5-7 days/week | 15 mg/kg/day; 5-7 days/week- |
| *Holtz (Van der Walt) [27]* | 800-1200 mg/day | 1000-2000 mg/day | 600-800 mg/day | Not used | Not used- | 750-1000 mg/day | 750-1000 mg/day | | Not used | Not used- |
| *DH Kim (Shim) [28]* | 800-1200 mg/day | 1000-2000 mg/day | 600-800 mg/day | 500 mg/day | 400mg/day | 15 mg/kg/day (max. 1.0 g) | 15 mg/kg/day (max. 1.0 g) | | 15 mg/kg/day (max. 1.0 g) | 15 mg/kg/day (max. 1.0 g) |
| *HR Kim (Yim) [29]* | 800-1200 mg/day | 1000-2000 mg/day | 600-800 mg/day | 500mg/day | 400mg/day | 15 mg/kg/day (max. 1.0 g) | 750-1000mg/day | | 750-1000mg/day | 750-1000mg/day |
| *Kwon (Koh) [30]* | 15-25 mg/kg/day  (max. 1.2 g) | 20-30 mg/kg/day (max. 2.0 g) | 600-800 mg/day | 500 mg/day | 400 mg/day | - | 15 mg/kg/day (max. 1.0 g) | | 15 mg/kg/day (max. 1.0 g) | 15 mg/kg/day (max. 1.0 g) |
| *Leimane/Holtz/Riekstina [31–33]* | 1200-1600 mg/day | 1500-2000 mg/day | 800 mg/day | Not used | 400 mg/day | 1000 mg/day | 1000 mg/day | | Not used | 1000 mg/day |
| *Masjedi (Tabarsi) [35]* | 15-25 mg/kg/day | 20-30 mg/kg/day | 400-800 mg/day | Not used | - | 15 mg/kg/day (max. 1.0 g) | - | | - | - |
| *Migliori (Centis) [36]* | 25 mg/kg/day  (max. 2.5 g) | 30-40 mg/kg/day (max. 2.5 g) | 15-20 mg/kg/day (max. 1.0 g) | 7.5-10 mg/kg/day (max. 1.0 g) | 7.5-10 mg/kg/day (max. 0.4 g) | 15-20 mg/kg/day (max. 1.0 g) | 15-20 mg/kg/day (max. 1.0 g) | | 15-20 mg/kg/day (max. 1.0 g) | 15-20 mg/kg/day (max. 1.0 g) |
| *Mitnick (Mitnick) [38,39]* | 25 mg/kg/day  (max. 2 g) | 30-40 mg/kg/day (max. 2.5 g) | 800 mg/day | 750 mg/day | 400 mg/day | 15-20 mg/kg/day (max. 1.0 g) | 15-20 mg/kg/day  (max. 1.0 g) | | 15-20 mg/kg/day  (max. 1.0 g) | 15-20 mg/kg/day (max. 1.0 g) |
| *Munsiff/Li (Ahuja)[40,41]* | 15-25 mg/kg  (max 2.5 g) | 1.5 g (<50 kg)  2.0 g (21-74 kg)  2.5 g (75+ kg) | 600-800 mg/day | 500 / day | Not used | 15 mg/kg/day | 15 mg/kg/day | | 15 mg/kg/day | 15 mg/kg/day |
| *Narita (Narita)[42]* | 15-25 mg/kg/d | 25 mg/kg/day  (max. 2.0 g) | 400-800 mg/day | 500 mg/day | Not used | 15-30 mg/kg/day  (max. 1.0 g | 15-30 mg/kg/day  (max. 1.0 g) | | 15 mg/kg/day  (max. 1.0 g) | 15 mg/kg/day  (max. 1.0 g) |
| *O’Riordan (Pasvol)[43]* | 15mg/kg/d | 25 mg/kg/day | Not used | Not used | 400 mg/d | 15-20mg/kg/day (max 1 gm) | Not used | | Not used | Not used- |
| *Palmero (Palmero)[44]* | - | 25 mg/k/day | 10 mg/kg/day (400-600 mg/day) | Not used | Not used | Not used | 15 mg/kg/day (max. 1.0 g) | | 15 mg/k/d (max. 1.0 g) | 15 mg/kg/day (max. 1.0 g) |
| *Park (Seung)[45]* | 800-1200 mg/day | 1.5-2.0 g/day | 150-300 mg, BID | Not used | Not used | Not used | 6.0 g/week for 2 months, 3.0 g/week for 2 months, then 2.0 g/week for 2 months intramuscularly | | 6.0 g/week for 2 months, 3.0 g/week for 2 months, then 2.0 g/week for 2 months intramuscularly | Not used- |
| *Perez-Guzman (Vargas) [46]* | 1200 mg/day | 2.0 g/day | 800 mg/day | Not used | Not used- | 1.0 g/day | 1.0 g/day | | 1.0 g/day | *Not used* |
| *Quy (Dang/Cobelens) [47]* | 15-20 mg/kg/d | 20-25 mg/kg/d | Not used | Not used | Not used | Not used | Not used | |  | Not used |
| *Schaaf (Schaaf)[48]* | 15-25 mg/kg/day | 25-35 mg/kg/day | 10-15 mg/kg/day | Not used | Not used | 15 mg/kg/day | 15 mg/kg/day | | 15-20 mg/kg/day | Not used |
| *Shin (Shin)[49]* | 25 mg/kg/day  (max. 2.5 g) | 30-40 mg/kg/day (max. 2.5 g) | 15-20 mg/kg/day (max. 1.0 g) | 7.5-10 mg/kg/day (max. 1.0 g) | 7.5-10 mg/kg/day (max. 400 mg) | 15-20 mg/kg/day (max. 1.0 g) | 15-20 mg/kg/day (max. 1.0 g) | | 15-20 mg/kg/day (max. 1.0 g) | 15-20 mg/kg/day (max. 1.0 g) |
| *Shiraishi (Shiraishi) [50]* | 750 mg/day | 1.5 g/day | 300-600 mg/day | 300-500 mg/ day | 400 mg/day | Not used | 0.75-1.0 g/day | | 0.75-1.0 g/day | Not used |
| *Tupasi (Quelapio) [51,52]* | 25 mg/kg/day (max. 2 g) | 30-40 mg/kg/day  (max 2.5 g) | 15-20 mg/kg/day  (max 1 g) | 7.5-10 mg/kg/day (max 1 g) | 7.5-10 mg/kg/day  (max 400 mg) | 15-20 mg/kg/day (max 1 g) | 15-20 mg/kg/day (max 1 g) | | 15-20  mg/kg/day (max 1 g) | 25 mg/kg/day (max. 2 g) |
| *Uffredi (Robert) [53]* | 10-25 mg/Kg/day | 20-35 mg/kg/day | 200-800 mg/day | Not used | Not used | 750-1500 mg/day | | | 1000 mg/day | 500-750 mg/day- |
| *Van Deun (Aung Maug) [54,55]* | 800 -1200 mg/day | 1000-2000 mg/day | 400-800 mg/day | Not used | 400-800 mg/d (Gatiflox) | 15-20 mg/kg/day | | | Not used | 1000 mg/day† |
| *Yew (Leung)[56,57]* | 15-25 mg/kg/day | 1.5-2.0 g/day | 600-800 mg/day | 600-800 mg/day | Not used | 15mg/kg/day (max. 0.75 g) | | 15mg/kg/day (max. 0.75 g) | 15mg/kg/day (max. 0.75 g) | 15mg/kg/day (max. 0.75 g) |
|  |  |  |  |  |  |  | |  |  |  |

* First-line treatment regimen only.

† One patient only.
