# Supplementary material for: Multidrug Resistant Pulmonary Tuberculosis Treatment Regimens and Patient Outcomes: An Individual Patient Data Meta-analysis of 9,153 Patients
Source: PLoS Med. 2012 Aug 28;9(8):e1001300. doi: 10.1371/journal.pmed.1001300 (PMC3429397; doi:10.1371/journal.pmed.1001300)
Supplement: Table S3 — Summary of treatment outcome definitions used in studies included in individual patient data meta-analysis. (DOC) [file pmed.1001300.s011.doc]

**Supplemental Table 3: Summary of treatment outcome definitions used in studies included in Individual Patient Data Meta-analysis**

| **First Author (Contact Person)**  **Country** | **Cure** | **Treatment Completed** | **Treatment Default** | **Treatment Failure** | **Treatment Relapse** |
| --- | --- | --- | --- | --- | --- |
| ***WHO & Laserson Criteria***  ***(Reference Standard)*** | *Completed treatment according to country protocol and has at least 5 consecutive negative cultures from samples collected at least 30 days apart in the final 12 months of treatment.*  *Exception: If only one positive culture is reported during that time, and there is no concomitant clinical deterioration, a patient can be considered cured, provided positive culture is followed by minimum of three consecutive negative cultures, taken at least 30 days apart.* | *Completed treatment but does not meet definition for cure or treatment failure due to lack of bacteriologic results (i.e., fewer than 5 cultures were performed in the final 12 months of therapy)* | *MDR-TB treatment interrupted for 2 or more consecutive months for any reason* | *Failed if two or more of five cultures recorded in final 12 months are positive, or if any one of the final three cultures is positive. OR clinical decision has been made to terminate treatment early due to poor response or adverse events.* | *Not defined* |
| ***Avendaño***  *Canada* | Negative culture for 24 months after bacteriologic conversion  *Bacteriological Conversion*  occurs if three sets of negative cultures of three sputum specimens were obtained on three consecutive days, each set being separated by one month.  *Criteria Categorization:*  Similar | Completed treatment  *Criteria Categorization:*  Similar | *Criteria Categorization:*  Not Available | *Criteria Categorization****:***  Not Available | Positive culture *for M. tuberculosis* two years after persistently negative cultures.  *Criteria Categorization****:***  Similar |
| ***Burgos***  *USA (San Francisco)* | Finished prescribed course of treatment with a combination of the drugs to which the isolate was susceptible with a microbiological  response and clinical evidence of resolution  of symptoms.  *Criteria Categorization:*  Similar | Not included in dataset.  *Criteria Categorization:*  Not Available | Stopped therapy before cure.  *Criteria Categorization:*  Similar | Culture results remained positive.  *Criteria Categorization:*  Similar | Reoccurrence of a positive culture result after having been considered cured.  *Criteria Categorization*:  Similar |
| ***Chan (Strand)***  *USA (Denver)* | Patients with at least 3 consecutive negative sputum cultures over a period of at least 3 months while on treatment.  (termed ‘initial favourable response’)  *Criteria Categorization:*  Similar | Not included in dataset.  *Criteria Categorization:*  Not Available | Not included in dataset.  *Criteria Categorization:*  Not Available | Patients who failed to achieve three consecutive negative sputum cultures over at least a 3-month period.  *Criteria Categorization:*  Reference Standard | Patients with ‘initial favourable response’ who became culture positive.  *Criteria Categorization*  *Similar* |
| ***Chiang***  ***(Enarson)***  *Taiwan* | Culture negative and documented to remain culture negative ≥1 month later, and never documented to become positive again up to 18 months after commencing treatment.  *Criteria Categorization:*  Reference Standard | Not included in the dataset.  *Criteria Categorization:*  Not available | Interrupted treatment for ≥2 months before the planned completion of treatment.  *Criteria Categorization:*  Reference Standard | Remained positive or became positive again ≥12 months after commencing the course of treatment.  *Criteria Categorization:*  Reference Standard | Patients considered eligible for relapse if cured at 18 months following commencement of treatment. Patient judged to have relapsed if they presented again as bacteriologically positive.  *Criteria Categorization*:  Similar |
| ***Cox***  *Uzbekistan* | At least 5 negative sputum cultures in the last 12 months of treatment. A single positive culture was allowed if it was followed by 3 negative cultures.  *Criteria Categorization:*  Reference Standard | Insufficient bacteriological results to classify the patient as cured, but no evidence of treatment failure.  *Criteria Categorization:*  Reference Standard | An interruption of two or more consecutive months to treatment.  *Criteria Categorization:*  Reference Standard | Two or more positive cultures in the last 12 months of treatment, or if a medical decision was made to terminate treatment due to poor response or adverse events.  *Criteria Categorization:*  Reference Standard | Not defined in paper.  *Criteria Categorization:*  Reference Standard |
| ***DeRiemer (Garcia)***  *Mexico* | Resolution of signs and symptoms at the completion of therapy (cure was considered bacteriologically confirmed if results were negative)  *Criteria Categorization:*  Similar | Not included in paper.  *Criteria Categorization:*  Not Available | Attended less than 80% of planned appointments, clinical consultations or laboratory controls, or who stopped going to the clinic for >=60 days.  (Includes WHO definition + overall measure of treatment compliance)  *Criteria Categorization:* Similar | Patients with positive AFB smear or culture results at the fifth month of treatment.  *Criteria Categorization:*  Reference Standard | Reappearance of bacilli in sputa after cure.  *Criteria Categorization*: Similar |
| ***Escudero (Pena)***  *Spain* | Last 2 AFB smears AND culture negative  *Criteria Categorization*  Similar | Planned treatment  completed, without meeting cure criteria  *Criteria Categorization*  Similar | Treatment was interrupted for 2 or mor consecutive months  *Criteria Categorization:*  Reference Standard | Positive cultures after 5 months or later during treatment  *Criteria Categorization:*  Reference Standard | Not included in dataset |
| ***Geerligs*** *(van der Werf)*  *Netherlands* | No clinical or microbiological signs of TB at the moment of follow-up. Follow-up for two years  *Criteria Categorization:*  Similar | Not defined in paper.  *Criteria Categorization:*  Unknown | Not defined in paper.  *Criteria Categorization:*  Unknown | 1 positive culture after 5 months of adequate therapy (see paper for adequate therapy)  *Criteria Categorization:*  Unknown | Not defined in paper.  *Criteria Categorization:*  Unknown |
| ***Granich and Banerjee*** *(Flood)*  *USA* | culture conversion documented    *Criteria Categorization:*  Similar | 18-24 months of MDR-TB treatment completed (usually with evidence of culture conversion and clinical improvement)  *Criteria Categorization:*  Reference standard | Did not complete adequate treatment as defined by local TB Controller  *Criteria Categorization:*  Similar | None  *Criteria Categorization*:  Not available | Not included in the dataset  *Criteria Categorization*:  Not available |
| ***Holtz*** *(Van der Walt)*  *S Africa* | Patient completed treatment, minimum of 5 cultures performed in the last 12 months of treatment with a maximum of only 1 positive culture during that time  *Criteria Categorization:*  Similar | Completed treatment in 22 months and does not meet definition for cure or failure due to lack of bacteriological results  *Criteria Categorization:*  Reference Standard | MDR-TB treatment interrupted for 2 or more consecutive months followed by a positive culture  *Criteria Categorization:*  Reference Standard | More than one positive culture in the last 12 months of treatment with minimum of 5 cultures performed during the 12 months or if one of the last 2 cultures is positive or stay culture positive  *Criteria Categorization:*  Reference Standard | Not included in dataset |
| ***DH Kim*** *(Shim****)***  *South Korea* | WHO-Laserson Criteria  *Criteria Categorization:*  Reference Standard | “Short-term treatment completion” defined as:  (1) inadequate treatment duration but duration of more than 6 months (2) more than three consecutive negative cultures before treatment completion and (3) treatment completion by a doctor based on favourable treatment response.  “Adequate treatment” defined as 18 months or more and 12 months or more after culture conversion.  *Criteria Categorization:*  Reference Standard | WHO-Laserson Criteria  *Criteria Categorization:*  Reference Standard | WHO-Laserson Criteria  *Criteria Categorization:*  Reference Standard | Not included in dataset.  *Criteria Categorization:*  Not Available |
| ***HR Kim*** *(Yim)*  *South Korea* | WHO-Laserson Criteria  *Criteria Categorization:*  Reference Standard | WHO-Laserson Criteria  *Criteria Categorization:*  Reference Standard | WHO-Laserson Criteria  *Criteria Categorization:*  Reference Standard | WHO-Laserson Criteria  Treatment-failure group includes patients with “relapse” or “failure”  *Criteria Categorization:*  Reference Standard | If a cured patient or patient who completed therapy resumed treatment >6months after completion of the first treatment because of the emergence of MDR-tuberculosis bacilli.  *Criteria Categorization:* Similar |
| ***Kwon*** *(Koh)*  *South Korea* | Completed treatment and consistently negative culture results (with at least 5 negative results) during final 12 months of treatment.  *Criteria Categorization:*  Reference Standard | Patients who completed treatment but did not meet definition for cure or experienced treatment failure.  *Criteria Categorization:*  Reference Standard | Did not receive treatment for >=2 consecutive months  (WHO definition)  *Criteria Categorization:*  Reference Standard | >=2 positive culture results recorded during final 12 months or a positive result for any one of the final three cultures.  *Criteria Categorization:*  Reference Standard | Not included in dataset.  *Criteria Categorization:*  Not available |
| ***Leimane/Riekstina***  *Latvia* | Patient who completed treatment (18 months after culture conversion) and has at least five consecutive negative cultures from samples collected at least 30 days apart in the final 12 months of treatment  *Criteria Categorization:*  Reference Standard | Patient who completed treatment, but does not meet the definition for cure because of lack of bacteriological results  *Criteria Categorization:*  Reference Standard | If treatment was interrupted for two or more consecutive months  *Criteria Categorization:*  Reference Standard | If two or more of the final five cultures recorder in the final 12 months of therapy are positive, or if any one of the final three cultures is positive  *Criteria Categorization:*  Reference Standard | *NA* |
| ***Masjedi*** *(Tabarsi)*  *Iran* | Patient has completed treatment and is consistently culture-negative, with at least five negative results in the last 12 months of treatment OR no clinical deterioration with at most one positive culture followed by three negative cultures 30 days apart in the final 12 months.  *Criteria Categorization:*  Reference Standard | Patient completed treatment but cannot be categorized as cure or treatment failure due to lack of adequate mycobacteriological evidence.  *Criteria Categorization:*  Reference Standard | Not included in the dataset.  *Criteria Categorization:*  Not Available | Two or more positive cultures among the five cultures in the final 12 months of treatment OR any positive culture in the last three cultures OR premature treatment termination due to poor response or adverse effects.  *Criteria Categorization:*  Reference Standard | Not included in dataset.  *Criteria Categorization*:  Not Available |
| ***Migliori*** *(Centis)*  *Italy* | Sputum smears were negative on two occasions at the end of treatment or in the presence of a documented culture conversion during the continuation phase.  *Criteria Categorization****:***  Reference Standard | Documented treatment completion but no sputum smear microscopy/culture conversion at the end of treatment.  *Criteria Categorization:*  Reference standard | Not defined in the paper but references WHO & IUATLD documents  *Criteria Categorization:*  Reference Standard | Not included in the dataset.  *Criteria Categorization:*  Not available | All cases who suffered a new episode of TB after having been considered “cured” or after having completed a previous treatment regimen for the first disease episode.  *Criteria Categorization*:  Similar |
| ***Mitnick***  *Peru* | WHO-Laserson Criteria  *Criteria Categorization:*  Reference Standard | WHO-Laserson Criteria  *Criteria Categorization:*  Reference Standard | WHO-Laserson Criteria  *Criteria Categorization:*  Reference Standard | WHO-Laserson Criteria  *Criteria Categorization:*  Reference Standard | Not included in the dataset.  *Criteria Categorization:*  Not Available |
| ***Munsiff/Li (****Ahuja****)***  *USA (NYC)* | Not included (outcome not assessed in jurisdiction) | At least 18 months of MDR-TB treatment with at least 12 months of treatment following the last negative culture  *Criteria Categorization:*  Similar | Any patients that were lost, refused treatment, moved, or did not complete treatment for any reason, except death  *Criteria Categorization:*  Similar | Patients with a positive culture ≥ 5 months after starting MDR-TB treatment, regardless of site of disease  *Criteria Categorization:*  Similar | A positive culture after treatment completion  Criteria Categorization:  Similar |
| ***Narita*** *(Ashkin)*  *USA (Florida)* | Documented treatment with at least two drugs to which the strain of M. tuberculosis is known to be susceptible, for at least 12 months after culture conversion.  *Criteria Categorization:*  similar | Documented treatment with at least two drugs to which the strain *of M. tuberculosis* is known to be susceptible, for at least 12 months after culture conversion.  *Criteria Categorization:*  *Similar* | Not included in dataset.  *Criteria Categorization:*  Not available | Not included in dataset.  *Criteria Categorization:*  Not available | Not included in dataset.  *Criteria Categorization:*  Not available |
| ***O’Riordan*** *(Pasvol****)***  *UK (London)* | Clinical with or without confirmed microbiological resolution of the disease without relapse of symptoms or positive culture.  *Criteria Categorization*  *Not similar* | Not included in dataset.  *Criteria Categorization:*  Not Available | Outcome included as “loss to follow-up” but not defined in papers.  *Criteria Categorization:*  Not Available | Outcome included but not defined in papers.  *Criteria Categorization:*  Not Available | Outcome included but not defined in papers.  *Criteria Categorization:*  Not Available |
| ***Palmero***  *Argentina* | Patients under treatment with effective drugs for 1 year since their first negative sputum culture and with at least five negative cultures during last year of treatment.  *Criteria Categorization:*  Reference Standard | Not included in dataset.  *Criteria Categorization:*  Not Available | More than a month’s absence from weekly medical appointments.  *Criteria Categorization:*  Reference Standard | Patients still on treatment at the end of the follow-up period with positive or negative sputum culture,  *Criteria Categorization:*  Reference Standard | Two positive cultures after treatment completion.  *Criteria Categorization*: Similar |
| ***Park*** *(Seung)*  *South Korea* | Completed treatment, and consistently culture-negative during the last 18 months of treatment  *Criteria Categorization:*  Reference Standard | Not included in dataset.  *Criteria Categorization:*  Not Available | Interruption of treatment for 2 or more consecutive months  *Criteria Categorization:*  Reference Standard | Persistently culture-positive after 6 months  *Criteria Categorization:*  Reference Standard | Sputum-positive after treatment of therapy with confirmation of bacteriological cure.  *Criteria Categorization:* Similar |
| ***Perez-Guzman*** *(Vargas)*  *Mexico* | Sputum culture negative at the end of 12-month treatment period.  *Criteria Categorization:*  Similar | Not included in dataset.  *Criteria Categorization:*  Not Available | Patients who stopped attending the clinic (and stopped treatment for more than 2 months) for unknown reasons after the first, second or third visit after initiating treatment.  *Criteria Categorization:*  Similar | Sputum culture positive at the end of 12-month treatment period.  *Criteria Categorization:*  Similar | Patients who were classified cure and who six months after end of treatment had positive sputum culture.  *Criteria Categorization:*  Similar |
| ***Quy*** *(Dang/Cobelens)*  *Vietnam* | Patient who is sputum smear negative in the last month of treatment and on at least one previous occasion.  *Criteria Categorization:*  Similar (smears) | Patient who has completed treatment but does not meet the criteria to be classified as cure or failure.  *Criteria Categorization:*  Reference Standard | Patient whose treatment was interrupted for two consecutive months or more.  *Criteria Categorization:*  Reference Standard | Patient who is sputum smear positive at five months or later during treatment.  *Criteria Categorization:*  Reference Standard | Not included in dataset.  *Criteria Categorization:*  Not Available |
| *Schaaf*  *South Africa* | two negative cultures and the child being clinically well  *Criteria Categorization:*  Similar | Child clinically well at end of treatment but not 2 neg cultures  *Criteria Categorization:*  Similar | if patient did not take any further treatment despite recall before end of treatment,  *Criteria Categorization:* Similar | culture positive at end of treatment...  *Criteria Categorization:*  Similar | Outcome included but not defined in papers.  *Criteria Categorization:*  Not available |
| ***Shin***  *Russia* | WHO-Laserson Criteria  *Criteria Categorization:*  Reference Standard | WHO-Laserson Criteria  *Criteria Categorization:*  Reference Standard | WHO-Laserson Criteria  *Criteria Categorization:*  Reference Standard | WHO-Laserson Criteria  *Criteria Categorization:*  Reference Standard | *Criteria Categorization:*  Not Available |
| ***Shiraishi***  *Japan* | From correspondence  *Criteria Categorization:*  Similar | Not included in dataset.  *Criteria Categorization:*  Not Available | Not included in dataset.  *Criteria Categorization:*  Not Available | Not included in dataset.  *Criteria Categorization:*  Not Available | Not included in dataset.  *Criteria Categorization:*  Not Available |
| ***Tupasi***  *Phillippines* | Five culture negatives in the last 12 months. If positive once, this had to be followed by 3 consecutive negative cultures taken 30 days apart and no clinical deterioration  *Criteria Categorization:*  Reference Standard | Fewer than five cultures in the last 12 months (all negative for those performed)  *Criteria Categorization:*  Reference Standard | Patient has not taken treatment for 2 consecutive months  *Criteria Categorization:*  Reference Standard | Two or more of the five cultures in the last 12 months are positive OR at least one culture positive in the final 3 cultures  *Criteria Categorization:*  Reference Standard | *NA* |
| ***Uffredi*** *(Robert)*  *France* | Last culture done is negative and clinical improvement and radiological improvement  *Criteria Categorization:*  Similar | Completed the entire course of treatment for the duration planned, clinical improvement and no microbiological sample  *Criteria Categorization:*  Reference Standard | Lost to follow up before treatment completion of the duration planned.  *Criteria Categorization:* Similar | Culture positive during the minimal 18 months follow- up after at least one negative culture and no clinical improvement  *Criteria Categorization:*  Reference Standard | Not included in dataset |
| ***Van Deun*** *(Aung Maug)*  *Bangladesh* | Completing the full course of treatment with sputum-smear negative on at least two to three occasions spanning a period of at least 6 months, with of one of these samples being taken at the foreseen end of treatment.  *Criteria Categorization:* Similar | Completed the treatment course, but incomplete documentation by sputum smears according to criteria for ‘cure’.  *Criteria Categorization:*  Reference Standard | Patients who refuse to continue treatment, after an interruption of at least two months and repeated home visits.  *Criteria Categorization:*  *Reference Standard* | Negative sputum cultures never obtained or only temporarily, or if a sputum culture is positive less than a month after end of treatment. If cultures of one of the last two samples positive, two more specimens sent - and if one was positive, outcome was “failure”  *Criteria Categorization:*  *Reference Standard* | Two positive sputum-specimens at least one month apart in cases who completed treatment or formerly declared cured, and more than one month after end of treatment. Relapse confirmed with IS6110 RFLP.  *Criteria Categorization:*  Reference standard |
| ***Yew*** *(Leung)*  *Hong Kong* | Sustained bacteriologic conversion of sputum culture of AFB from positive to negative for at least 6 consecutive months during therapy and after its cessation.  *Criteria Categorization:*  Reference Standard | Not included in dataset.  *Criteria Categorization:*  Not Available | Patients who refuse to continue treatment, after an interruption of at least two months and repeated home visits.    *Criteria Categorization:*  Reference Standard | Absence of conversion to negative throughout treatment. or temporary conversion not meeting the “Cure” criteria.  (Semi-WHO definition)  *Criteria Categorization:*  Reference Standard | Not included in dataset.  *Criteria Categorization:*  Not Available |

Criteria categorization for Treatment Outcomes: Reference Standard, Similar, Other, Not Available
