# Supplementary material for: Multidrug Resistant Pulmonary Tuberculosis Treatment Regimens and Patient Outcomes: An Individual Patient Data Meta-analysis of 9,153 Patients
Source: PLoS Med. 2012 Aug 28;9(8):e1001300. doi: 10.1371/journal.pmed.1001300 (PMC3429397; doi:10.1371/journal.pmed.1001300)
Supplement: Table S4 — Characteristics of patients associated with history of prior TB therapy. (DOC) [file pmed.1001300.s012.doc]

**Supplemental Table 4: Characteristics of patients associated with history of prior TB therapy*.**

|  | **No prior therapy** | **Prior TB therapy**** | | **P value***** |
| --- | --- | --- | --- | --- |
|  |  | **First line drugs** | **Second-line drugs** |  |
| Total Number | 2027 | 5735 | 942 |  |
| Demographic characteristics |  |  |  |  |
| Age (Mean, SD) | 39.6 (14.2) | 38.8 (13.4) | 36.0 (12.5) | .01 |
| Sex - Male (N, %) | 1362 (67%) | 3981 (69%) | 620 (66%) | .09 |
|  |  |  |  |  |
| Clinical characteristics (N, %) |  |  |  |  |
| AFB – Smear positive | 1230 (77%) | 3912 (77%) | 673 (78%) | <.001 |
| Cavities on x-ray | 644 (58%) | 3036 (70%) | 704 (87%) | <.001 |
| Extensive disease | 1458 (74%) | 4291 (76%) | 736 (78%) | <.001 |
| HIV positive | 483 (28%) | 551 (11%) | 12 (1%) | <.001 |
| Pulmonary only | 1936 (98%) | 5526 (98%) | 859(95%) | <.001 |
|  |  |  |  |  |
| Resistance to other first line drugs (N,%) |  |  |  |  |
| Ethambutol | 1114 (64%) | 2171 (56%) | 524 (69%) | <.001 |
| Pyrazinamide | 751 (49%) | 1194 (48%) | 440 (66%) | <.001 |
| Streptomycin | 1075 (62%) | 2264 (61%) | 526 (75%) | <.001 |
|  |  |  |  |  |
| Treatment received (N, %) |  |  |  |  |
| Rifabutin | 41 (2%) | 27 (0.5%) | 50 (5%) | <.001 |
| Ethambutol | 1114 (57%) | 3106 (54%) | 287 (30%) | <.001 |
| Pyrazinamide | 1420 (70%) | 4362 (76%) | 399 (42%) | <.001 |
| Ciprofloxacin | 376 (19%) | 244 (4%) | 357 (38%) | <.001 |
| Ofloxacin | 1344 (66%) | 4646 (81%) | 469 (50%) | <.001 |
| Later generation quinolones | 56 (3%) | 517 (9%) | 250 (27%) | <.001 |
| Streptomycin | 404 (19%) | 707 (12%) | 157 (17%) | <.001 |
| Kanamycin/Amikacin | 812 (40%) | 3936 (69%) | 459 (49%) | <.001 |
| Capreomycin | 461 (23%) | 736 (13%) | 441 (47%) | <.001 |
| Ethionamide/prothionamide | 1341 (66%) | 4959 (86%) | 705 (75%) | <.001 |
| Cycloserine/terizidone | 1270 (63%) | 2993 (52%) | 809 (86%) | <.001 |
| Para-amino-salicylic acid (PAS) | 737 (36%) | 2048 (36%) | 709 (75%) | <.001 |
| Group 5 drugs (any) | 479 (23%) | 1488 (26%) | 618 (66%) | <.001 |

* Excludes – 443 patients in whom the past treatment history was unknown/missing

** Prior TB therapy defined as treatment with any TB drugs for one month or more. Prior MDR therapy defined as treatment with two or more second line drugs. In some patients information was available regarding prior TB therapy, but not whether this was with first or second line drugs.

*** P Value from Chi squared test - comparing characteristics in all 3 treatment groups

Later generation quinolones defined as levofloxacin, moxifloxacin, gatifloxacin, and sparfloxacin.

Group 5 drugs included amoxicillin-clavulanate, macrolides, clofazimine, thiacetazone, imipenem, linezolid, high dose INH, and thioridazine.
