# Supplementary material for: Multidrug Resistant Pulmonary Tuberculosis Treatment Regimens and Patient Outcomes: An Individual Patient Data Meta-analysis of 9,153 Patients
Source: PLoS Med. 2012 Aug 28;9(8):e1001300. doi: 10.1371/journal.pmed.1001300 (PMC3429397; doi:10.1371/journal.pmed.1001300)
Supplement: Table S5 — Association of Individual drugs with treatment success (compared to failure/relapse)—stratified by history of previous treatment. (DOC) [file pmed.1001300.s013.doc]

**Supplemental Table 5: Association of Individual drugs with Treatment Success (compared to failure/relapse) - stratified by history of previous treatment.**

|  | **All patients** | | | **No prior MDR treatment** | | | | **Prior MDR treatment** | | | |  |
| --- | --- | --- | --- | --- | --- | --- | --- | --- | --- | --- | --- | --- |
|  | N | aOR | (95%CI) | N | aOR | (95%CI) | | | N | aOR | (95%CI) | |
| **Group 1 Drugs** |  |  |  |  |  |  | | |  |  |  | |
| Pyrazinamide | 3985 | 1.2 | (0.9, 1.7) | 3739 | 1.2 | (0.9,1.4) | | | 246 | 1.4 | (0.6, 3.1) | |
| Ethambutol | 2819 | 0.9 | (0.7, 1.1) | 2639 | 0.9 | (0.7, 1.1) | | | 180 | 1.2 | (0.6, 2.4) | |
|  | | | | | | |  | | | | | |
| **Injectables** (patients receiving 2 or more injectables excluded from this analysis) | | | | | | | | |  |  |  | |
| Kanamycin only |  |  |  |  |  |  | | |  |  |  | |
| vs no injectable | 3535 | 1.0 | (0.5, 2.1) | 3337 | 1.1 | (0.6, 2.2) | | | 198 | 1.1 | (0.2, 4.8) | |
| vs Capreomycin | 3696 | 1.5 | (0.8, 3.0) | 3411 | 1.6 | (0.8, 3.2) | | | 285 | 1.1 | (0.3, 4.4) | |
| vs Streptomycin | 3535 | 1.2 | (0.6, 2.2) | 3315 | 1.1 | (0.6, 1.9) | | | 220 | 1.2 | (0.3, 5.3) | |
|  |  |  |  |  |  |  | | |  |  |  | |
| Capreomycin only |  |  |  |  |  |  | | |  |  |  | |
| vs no injectable | 1469 | 1.0 | (0.4, 2.4) | 1274 | 1.0 | (0.4, 2.6) | | | 195 | 1.4 | (0.1, 23) | |
| vs Streptomycin | 1281 | 0.7 | (0.3, 1.5) | 1085 | 0.6 | (0.2,1.4) | | | 196 | 1.0 | (0.1, 6.7) | |
|  | | | | | | |  | | | | | |
| **Quinolones** (patients receiving 2 or more Quinolones excluded from this analysis) | | | | | | | | |  |  |  | |
| Later generation Quinolones |  |  |  |  |  |  | | |  |  |  | |
| vs no Quinolones | 1169 | **2.5** | **(1.1, 6.0)** | 1058 | 2.4 | (0.9, 6.7) | | | 111 | 3.1 | (0.2, 62) | |
| vs Ofloxacin | 4583 | **1.7** | **(1.1, 2.7)** | 4297 | **1.8** | **(1.1, 2.8)** | | | 286 | 1.6 | (0.4, 6.9) | |
| Ofloxacin |  |  |  |  |  |  | | |  |  |  | |
| vs no Quinolones | 4250 | **2.5** | **(1.6, 3.9)** | 4025 | **2.6** | **(1.7, 1.10** | | | 225 | 1.5 | (0.3, 6.4) | |
| vs Ciprofloxacin | 4167 | 1.1 | (0.5, 2.5) | 3845 | 1.1 | (0.5, 2.5) | | | 322 | 0.2 | (0.1, 0.8) | |
|  |  |  |  |  |  |  | | |  |  |  | |
| **Group 4 Drugs** |  |  |  |  |  |  | | |  |  |  | |
| Ethionamide/Prothionamide | 4608 | **1.7** | **(1.3, 2.3)** | 4173 | **1.6** | **(1.2, 2.1)** | | | 435 | **1.8** | **(1.0, 3.4)** | |
| Cycloserine/Terizidone | 3548 | 1.1 | (0.8, 1.7) | 3043 | 1.1 | (0.7, 1.7) | | | 505 | 1.0 | (0.3, 2.9) | |
| PAS | 2024 | 0.9 | (0.6, 1.4) | 1631 | 0.9 | (0.6, 1.4) | | | 393 | 1.0 | (0.4, 2.4) | |
| **Group 5 Drugs** (patients receiving 2 or more Group 5 drugs excluded from this analysis) | | | | | | | | |  |  |  | |
| Any 1 Group 5 vs none | 1603 | 0.6 | (0.4, 0.9) | 1431 | 0.6 | (0.4, 0.9) | | | 172 | 0.7 | (0.2, 2.2) | |
| Amox-Clavulanate only | 232 | 1.0 | (0.4, 2.5) | 160 | 0.6 | (0.2, 1.6) | | | 72 | 3.1 | (0.1, 66) | |
| Clofazimine only | 651 | 2.7 | (0.6,12.1) | 620 | 1.0 | (0.5, 1.7) | | | 31 | - | (---, ---) | |
| Macrolide only | 108 | 0.4 | (0.2, 1.1) | 99 | 0.9 | (0.3, 2.5) | | | 9 | - | (---, ---) | |
| Thiacetazone only | 541 | 0.9 | (0.1, 6.8) | 484 | 1.1 | (0.2, 7.8) | | | 57 | 0.3 | (0.1, 11) | |

Success: defined as cure or treatment completion and is compared to failure or relapse (see methods for definitions)

Prior TB therapy defined as treatment with any TB drugs for one month or more. Prior MDR therapy defined as treatment with two or more second line drugs

N for injectables and quinolones are for total number of patients included in the analysis

N for Group 1, Group 4 and Group 5 drugs are the number of patients that received the drug in question and were included in the analysis.

Group 5 individual drugs: Analysis restricted to patients who received only one Group 5 drug. Each single drug comparison made between patients who received only that Group 5 agent with patients who received any other single Group 5 drug.
