# Supplementary material for: Multidrug Resistant Pulmonary Tuberculosis Treatment Regimens and Patient Outcomes: An Individual Patient Data Meta-analysis of 9,153 Patients
Source: PLoS Med. 2012 Aug 28;9(8):e1001300. doi: 10.1371/journal.pmed.1001300 (PMC3429397; doi:10.1371/journal.pmed.1001300)
Supplement: Table S6 — Secondary analyses to assess impact of covariates on duration of therapy. (A) Duration analysis also adjusted for use of four drugs. (B) Analysis restricted: excluded patients who received two or more injectables, and/or had a serious adverse event to an injectable (204 patients in two studies treated with first-line drugs also excluded). (C) Restricted analyses: use of second-line drugs only. (D) Stratified analysis: by use of later generation quinolones: 204 patients receiving first-line drugs only excluded. (DOC) [file pmed.1001300.s014.doc]

**Supplemental Table 6: Secondary analyses to assess impact of covariates on duration of therapy:**

**Table 6A: Duration analysis also adjusted for use of 4 drugs:**

**Duration of initial intensive phase.**

| Initial  Duration  Months | All patients | Duration adjusted for 4 Drugs (PZA, Kanamycin, Later generation Quinolones & ETO/PTO) |  |
| --- | --- | --- | --- |

|  | N | aOR | (95%CI) | N* | aOR | (95% CI) | |
| --- | --- | --- | --- | --- | --- | --- | --- |
|  |  |  |  |  |  | |  |
| 1 - 2.5 | 308 | 1.0 | (reference) | 168 | 1.0 | (reference) | |
| 2.6 - 4.0 | 1406 | 1.2 | (0.5, .2.9) | 1406 | 1.2 | (0.7, 1.9) | |
| 4.1 - 5.5 | 481 | 2.4 | (1.3,. 4.3) | 481 | 2.3 | (1.2, 4.1) | |
| 5.6 - 7.0 | 377 | 3.7 | (1.9, 7.1) | 377 | 3.3 | (1.8, 6.3) | |
| 7.1 - 8.5 | 172 | 5.1 | (2.1, 12.7) | 172 | 4.6 | (1.9,11.3) | |
| 8.6 - 20 | 792 | 2.2 | (1.2, 3.9) | 792 | 2.0 | (1.1, 3.5) | |

**Total Duration of therapy**

| Total  Duration  (months) | All patients | Duration adjusted for 4 Drugs (PZA, Kanamycin, Later generation quinolones & ETO/PTO) |  |
| --- | --- | --- | --- |

|  | N | aOR | (95%CI) | N* | aOR | (95% CI) |  |  | |  | |
| --- | --- | --- | --- | --- | --- | --- | --- | --- | --- | --- | --- |
|  |  |  |  |  |  |  |  | |  | |  |
| 6.0 - 12.5 | 778 | 1.0 | (reference) | 638 | 1.0 | (reference) |  |  | |  | |
| 12.6 - 15.5 | 419 | 1.8 | (1.3, 2,7) | 415 | 1.8 | (1.2, 2.6) |  |  | |  | |
| 15.6 - 18.5 | 1700 | 4.2 | (1.9, 9.1) | 1700 | 4.1 | (1.9, 9.0) |  |  | |  | |
| 18.6 - 21.5 | 655 | 7.8 | (5.1, 11.9) | 655 | 8.0 | (5.2,12.2) |  |  | |  | |
| 21.6 - 24.5 | 553 | 7.1 | (4.6, 10.9) | 353 | 7.1 | (4.6,11.0) |  |  | |  | |
| 24.6 - 27.5 | 313 | 7.9 | (4.4, 13.9) | 313 | 7.9 | (4.5,14.0) |  |  | |  | |
| 27.6 – 30.5 | 160 | 3.8 | (1.9, 7.4) | 160 | 3.7 | (1.9, 7.3) |  |  | |  | |
| 30.6 - 36 | 89 | 1.7 | (0.9, 3.2) | 89 | 1.8 | (1.0, 3.3) |  |  | |  | |

N – Number of patients in subgroup of interest. Two studies with 200 patients receiving only first line drugs were excluded from analysis adjusted for use of four drugs

aOR; adjusted odds ratios - adjusted for age, sex, HIV co-infection, past TB treatment, past MDR treatment, and extent of disease

Success: defined as cure or treatment completion and is compared to failure or relapse (see methods for definitions). Other outcomes of death and default not assessed in this analysis because in some data sets shorter duration that was directly due to death or default could not be identified.

Past treatment: Prior MDR means past treatment for more than one month with two or more second line drugs. No prior MDR includes all other treatment history.

**Supplemental Table 6B: Analysis restricted: Excluded patients who received 2 or more injectables, and/or had a serious adverse event to an injectable:**

*(204 patients in 2 studies treated with first line drugs also excluded)*

**Duration of initial intensive phase.**

| Initial | All patients | Excluded if 2 or more injectables or adverse event from injectable |  |
| --- | --- | --- | --- |
| Duration |  |  |  |

months

|  | N | aOR | (95%CI) | N | aOR | (95%CI) |  |  |  |
| --- | --- | --- | --- | --- | --- | --- | --- | --- | --- |
|  |  |  |  |  |  |  |  |  |  |
| 1 - 2.5 | 308 | 1.0 | (ref) | 139 | 1.0 | (ref) |  |  |  |
| 2.6 - 4.0 | 1406 | 1.2 | (0.5, .2.9) | 1390 | 1.5 | (0.7, 3.3) |  |  |  |
| 4.1 - 5.5 | 481 | 2.4 | (1.3,. 4.3) | 468 | 2.9 | (1.5, 5.5) |  |  |  |
| 5.6 - 7.0 | 377 | 3.7 | (1.9, 7.1) | 374 | 4.1 | (2.1, 8.1) |  |  |  |
| 7.1 - 8.5 | 172 | 5.1 | (2.1, 12.7) | 162 | 6.4 | (2.4, 16.8) |  |  |  |
| 8.6 - 20 | 792 | 2.2 | (1.2, 3.9) | 684 | 2.6 | (1.4, 4.9) |  |  |  |

**Total Duration of therapy**

| Total  Duration  (months) | All patients | Excluded if 2 or more injectables or adverse event from injectable |  |
| --- | --- | --- | --- |

|  | N | aOR | (95%CI) | N | aOR | (95%CI) |  |  |  |
| --- | --- | --- | --- | --- | --- | --- | --- | --- | --- |
|  |  |  |  |  |  |  |  |  |  |
| 6.0 - 12.5 | 778 | 1.0 | (ref) | 614 | 1.0 | (ref) |  |  |  |
| 12.6 - 15.5 | 419 | 1.8 | (1.3, 2,7) | 390 | 1.6 | (1.1, 2.4) |  |  |  |
| 15.6 - 18.5 | 1700 | 4.2 | (1.9, 9.1) | 1612 | 3.6 | (1.7, 7.5) |  |  |  |
| 18.6 - 21.5 | 655 | 7.8 | (5.1, 11.9) | 604 | 7.6 | (4.8, 12.0) |  |  |  |
| 21.6 - 24.5 | 553 | 7.1 | (4.6, 10.9) | 491 | 8.6 | (5.3, 14.1) |  |  |  |
| 24.6 - 27.5 | 313 | 7.9 | (4.4, 13.9) | 272 | 7.9 | (4.2, 14.6) |  |  |  |
| 27.6 – 30.5 | 160 | 3.8 | (1.9, 7.4) | 140 | 4.3 | (2.0, 9.2) |  |  |  |
| 30.6 - 36 | 89 | 1.7 | (0.9, 3.2) | 74 | 1.6 | (0.8, 3.1) |  |  |  |

N – Number of patients in subgroup of interest.

aOR; adjusted odds ratios - adjusted for age, sex, HIV co-infection, past TB treatment, past MDR treatment, and extent of disease

Success: defined as cure or treatment completion and is compared to failure or relapse (see methods for definitions). Other outcomes of death and default not assessed in this analysis because in some data sets shorter duration that was directly due to death or default could not be identified.

Past treatment: Prior MDR means past treatment for more than one month with two or more second line drugs. No prior MDR includes all other treatment history.

Only 7 studies reported serious adverse events to second line injectables. 107 patients had an SAE to kanamycin or amikacin of whom 7 (7%) received a second injectable. 16 patients had a serious adverse event with capreomycin of whom 3 (19%) received a second injectable compared to 5% of all patients who received a second injectable. The majority of these events were hearing loss or disturbances of vestibular function.

**Supplemental Table 6C - Restricted analyses: Use of second line drugs only**

**Duration of initial intensive phase.**

| Initial | **All patients** | **Only if received second line drugs** |  |
| --- | --- | --- | --- |
| Duration |  | N=8953 |  |

months

|  | N | aOR | (95%CI) | N | aOR | (95%CI) |  |  |  |
| --- | --- | --- | --- | --- | --- | --- | --- | --- | --- |
|  |  |  |  |  |  |  |  |  |  |
| 1 - 2.5 | 308 | 1.0 | (ref) | 168 | 1.0 | (ref) |  |  |  |
| 2.6 - 4.0 | 1406 | 1.2 | (0.5, .2.9) | 1406 | 1.1 | (0.5, 2.7) |  |  |  |
| 4.1 - 5.5 | 481 | 2.4 | (1.3,. 4.3) | 481 | 2.2 | (1.2, 4.1) |  |  |  |
| 5.6 - 7.0 | 377 | 3.7 | (1.9, 7.1) | 377 | 3.3 | (1.7, 6.3) |  |  |  |
| 7.1 - 8.5 | 172 | 5.1 | (2.1, 12.7) | 172 | 4.6 | (1.9,11.6) |  |  |  |
| 8.6 - 20 | 792 | 2.2 | (1.2, 3.9) | 792 | 1.9 | (1.1, 3.5) |  |  |  |

**Total Duration of therapy**

(months)

| Total | All patients | **Only if received second line drugs** |  |
| --- | --- | --- | --- |
| duration |  |  |  |

|  | N | aOR | (95%CI) | N | aOR | (95%CI) |  |  |  |
| --- | --- | --- | --- | --- | --- | --- | --- | --- | --- |
|  |  |  |  |  |  |  |  |  |  |
| 6.0 - 12.5 | 778 | 1.0 | (ref) | 638 | 1.0 | (ref) |  |  |  |
| 12.6 - 15.5 | 419 | 1.8 | (1.3, 2,7) | 419 | 1.8 | (1.2, 2.6) |  |  |  |
| 15.6 - 18.5 | 1700 | 4.2 | (1.9, 9.1) | 1700 | 4.1 | (1.9, 9.1) |  |  |  |
| 18.6 - 21.5 | 655 | 7.8 | (5.1, 11.9) | 655 | 7.8 | (5.1, 11.9) |  |  |  |
| 21.6 - 24.5 | 553 | 7.1 | (4.6, 10.9) | 553 | 7.1 | (4.6, 10.9) |  |  |  |
| 24.6 - 27.5 | 313 | 7.9 | (4.4, 13.9) | 313 | 7.9 | (4.4, 13.9) |  |  |  |
| 27.6 – 30.5 | 160 | 3.8 | (1.9, 7.4) | 160 | 3.7 | (1.9, 7.3) |  |  |  |
| 30.6 - 36 | 89 | 1.7 | (0.9, 3.2) | 89 | 1.7 | (0.9, 3.2) |  |  |  |

N – Number of patients in subgroup of interest.

aOR; adjusted odds ratios - adjusted for age, sex, HIV co-infection, past TB treatment, past MDR treatment, and extent of disease

Success: defined as cure or treatment completion and is compared to failure or relapse (see methods for definitions). Other outcomes of death and default not assessed in this analysis because in some data sets shorter duration that was directly due to death or default could not be identified.

Past treatment: Prior MDR means past treatment for more than one month with two or more second line drugs. No prior MDR includes all other treatment history.

**Supplemental Table 6D: Stratified analysis: by use of later generation Quinolones:**

*204 patients receiving first line drugs only Excluded*

Duration of initial intensive phase.

| Initial | All patients | No Later Generation | Later Generation |
| --- | --- | --- | --- |
| Duration |  | Quinolone | Quinolone Used |

months

|  | N | aOR | (95%CI) | N | aOR | (95%CI) | N | aOR | (95%CI) |
| --- | --- | --- | --- | --- | --- | --- | --- | --- | --- |
|  |  |  |  |  |  |  |  |  |  |
| 1 - 2.5 | 308 | 1.0 | (ref) | 163 | 1.0 | (ref) | 5 | 1.0 |  |
| 2.6 - 4.0 | 1406 | 1.2 | (0.5, .2.9) | 1364 | 1.3 | (0.5, 3.0) | 42 | *Did* |  |
| 4.1 - 5.5 | 481 | **2.4** | **(1.3,. 4.3)** | 165 | **2.1** | **(1.1, 4.0)** | 316 | *not* |  |
| 5.6 - 7.0 | 377 | **3.7** | **(1.9, 7.1)** | 287 | **3.1** | **(1.6, 6.0)** | 90 | *converge* |  |
| 7.1 - 8.5 | 172 | **5.1** | **(2.1, 12.7)** | 126 | **5.2** | **(1.9, 14.6)** | 46 | *Adjusted or* |  |
| 8.6 - 20 | 792 | **2.2** | **(1.2, 3.9)** | 633 | **2.3** | **(1.3, 4.3)** | 159 | *Unadjusted* |  |
|  |  |  |  |  |  |  |  |  |  |

**Total Duration of therapy**

(months)

| Total | | All patients | Did not receive later generation | Received later generation |
| --- | --- | --- | --- | --- |
| duratio**n** |  | | Fluoro-quinolone | Quinolone |

|  | N | aOR | (95%CI) | N | aOR | (95%CI) | N | aOR | (95%CI) |
| --- | --- | --- | --- | --- | --- | --- | --- | --- | --- |
|  |  |  |  |  |  |  |  |  |  |
| 6.0 - 12.5 | 778 | 1.0 | (ref) | 342 | 1.0 | (ref) | 296 | 1.0 | (ref) |
| 12.6 - 15.5 | 419 | **1.8** | **(1.3, 2,7)** | 372 | **3.2** | **(2.0, 5.0)** | 47 | 1.2 | (0.4, 3.2) |
| 15.6 - 18.5 | 1700 | **4.2** | **(1.9, 9.1)** | 1634 | **4.4** | **(2.2, 9.1)** | 66 | 8.0 | (0.7, 88) |
| 18.6 - 21.5 | 655 | **7.8** | **(5.1, 11.9)** | 574 | **11.1** | **(6.8, 18.1)** | 81 | **4.7** | **(2.0, 11.2)** |
| 21.6 - 24.5 | 553 | **7.1** | **(4.6, 10.9)** | 424 | **11.0** | **(6.4, 18.7)** | 129 | **4.2** | **(1.9, 9.2)** |
| 24.6 - 27.5 | 313 | **7.9** | **(4.4, 13.9)** | 192 | **11.4** | **(5.5, 23.9)** | 121 | **5.6** | **(2.2, 14.1)** |
| 27.6 – 30.5 | 160 | **3.8** | **(1.9, 7.4)** | 74 | **3.6** | **(1.5, 8.5)** | 86 | **4.9** | **(1.6, 14.6)** |
| 30.6 - 36 | 89 | 1.7 | (0.9, 3.2) | 50 | 2.0 | (0.9, 4.6) | 39 | 1.5 | (0.6, 3.9) |

Later generation fluoro-quinolones were moxifloxacin, levofloxacin, gatifloxacin and sparfloxacin.

N – Number of patients in subgroup of interest.

aOR; adjusted odds ratios - adjusted for age, sex, HIV co-infection, past TB treatment, past MDR treatment, and extent of disease

Success: defined as cure or treatment completion and is compared to failure or relapse (see methods for definitions). Other outcomes of death and default not assessed in this analysis because in some data sets shorter duration that was directly due to death or default could not be identified.

Past treatment: Prior MDR means past treatment for more than one month with two or more second line drugs. No prior MDR includes all other treatment history.
