# Supplementary material for: Multidrug Resistant Pulmonary Tuberculosis Treatment Regimens and Patient Outcomes: An Individual Patient Data Meta-analysis of 9,153 Patients
Source: PLoS Med. 2012 Aug 28;9(8):e1001300. doi: 10.1371/journal.pmed.1001300 (PMC3429397; doi:10.1371/journal.pmed.1001300)
Supplement: Table S7 — Pooled outcomes—studies stratified by study level factors (event rates pooled across studies—study level random effects meta-analysis). (DOC) [file pmed.1001300.s015.doc]

**Supplement Table 7: Pooled outcomes – studies stratified by study level factors**

*(Event rates pooled across studies – study level random effects meta-analysis)*

| Study Factor |  | | **Pooled rates of Outcomes** | | | |
| --- | --- | --- | --- | --- | --- | --- |
|  | N Studies | N Subjects | **Cure**  % (95% CI) | **Failed**  % (95% CI) | **Died**  % (95% CI) | **Defaulted**  % (95% CI) |
| **Relapse** |  |  |  |  |  |  |
| Measured | 17 | 4516 | 59% (50, 69) | 8% (5, 10) | 9% (5, 13) | 18% (11, 25) |
| Not measured | 15 | 4637 | 64% (52, 76) | 5% (1, 8) | 8% (3,12) | 15% (8, 22) |
| **Treatment Design** |  |  |  |  |  |  |
| Standardized overall | 7 | 3174 | 47% (32, 62) | 14% (7, 21) | 9% (2, 16) | 21% (11, 32) |
| Standardized - First line dugs | 2 | 204 | 27% (0, 57) | 26% (0, 99) | 14 % (3, 26) | 22% (6, 38) |
| Standardized - Sec line drugs | 5 | 2970 | 56% (45, 66) | 9% (5, 14) | 7% (0, 14) | 20% (6, 35) |
| Individualized - Sec line drugs | 25 | 5981 | 65% (57, 73) | 5% (3, 7) | 8% (5, 11) | 15% (10, 21) |
| **Number of subjects** |  |  |  |  |  |  |
| < 50 in each study | 9 | 318 | 60% (42, 78) | 5% (0, 10) | 8% (0, 17) | 16% (6, 25) |
| > 50 in each study | 23 | 8835 | 62% (54, 70) | 7% (4, 9) | 8% (5, 11) | 17% (11, 23) |
| **National TB Incidence** |  |  |  |  |  |  |
| < 100/100,000 | 22 | 4537 | 60% (49, 71) | 5% (2, 8) | 8% (4, 12) | 17% (10, 24) |
| > 100/100,000 | 10 | 4616 | 64% (57, 71) | 9% (5, 12) | 9% (6, 13) | 15% (10, 20) |
|  |  |  |  |  |  |  |
