# Supplementary material for: Multidrug Resistant Pulmonary Tuberculosis Treatment Regimens and Patient Outcomes: An Individual Patient Data Meta-analysis of 9,153 Patients
Source: PLoS Med. 2012 Aug 28;9(8):e1001300. doi: 10.1371/journal.pmed.1001300 (PMC3429397; doi:10.1371/journal.pmed.1001300)
Supplement: Table S8 — (a) Assessment of potential confounding of clinical characteristics and drug resistance with initial duration (only patients analyzed for success versus fail/relapse). (B) Assessment of potential confounding of clinical characteristics with total duration of therapy (only patients analyzed for success versus fail/relapse). (DOC) [file pmed.1001300.s016.doc]

Table 8a: Assessment of potential confounding of clinical characteristics and drug resistance with initial duration (only patients analyzed for success versus fail/relapse)

|  | 1-2.4  months | 2.5-3.9  months | 4.0-5.4  months | 5.5-6.9  months | 7.0-8.4  months | 8.5-10  months |
| --- | --- | --- | --- | --- | --- | --- |
| **Clinical characteristics** |  |  |  |  |  |  |
| Age (mean, years) | 38 | 36 | 35 | 41 | 37 | 35 |
| Sex (% male) | 66% | 64% | 71% | 68% | 65% | 70% |
| No prior TB Therapy | 30% | 9% | 8% | 24% | 19% | 5% |
| Prior First line TB drugs | 68% | 90% | 87% | 67% | 66% | 64% |
| Prior Second line TB drugs | 2% | 2% | 5% | 10% | 15% | 31% |
| Extensive disease | 81% | 73% | 84% | 84% | 74% | 76% |
| HIV (% pos) | 23% | 29% | 11% | 6% | 10% | 4% |
| **Drug Susceptibility tests** |  |  |  |  |  |  |
| PZA resistant | 49% | 51% | 48% | 48% | 55% | 51% |
| EMB resistant | 43% | 57% | 62% | 58% | 67% | 65% |
| FQN resistant | 4% | 2% | 10% | 15% | 10% | 10% |
| Streptomycin resistant | 65% | 68% | 72% | 58% | 72% | 80% |
| Kanamycin resistant | 13% | 5% | 8% | 22% | 24% | 27% |

Table B: Assessment of potential confounding of clinical characteristics with total duration of therapy (only patients analyzed for success vs fail/relapse.)

|  | 6.0-12.4  months | 12.5-15.4  months | 15.5-18.4  months | 18.5-21.4  months | 21.5-24.4  months | 24.5-27.4  months | 27.5-30.4  months | 30.5-36.0  months |
| --- | --- | --- | --- | --- | --- | --- | --- | --- |
| N | 778 | 419 | 1700 | 655 | 553 | 313 | 160 | 89 |
| **Clinical characteristics** |  |  |  |  |  |  |  |  |
| Prior First line drugs | 78% | 70% | 82% | 69% | 58% | 44% | 41% | 24% |
| Prior Second line drugs | 5% | 10% | 3% | %7 | 22% | 35% | 36% | 48% |
| Extensive disease | 84% | 77% | 67% | 73% | 77% | 77% | 79% | 71% |
| HIV (% pos) | 1% | 1% | 21% | 7% | 2% | 2% | 3% | 6% |
| **Drug Susceptibility tests** | |  |  |  |  |  |  |  |
| PZA resistant | 53% | 57% | 49% | 57% | 56% | 6% | 62% | 62% |
| EMB resistant | 52% | 67% | 53% | 63% | 67% | 69% | 69% | 70% |
| FQN resistant | 10% | 8% | 3% | 5% | 10% | 7% | 17% | 17% |
| Kanamycin resistant | 20% | 26% | 21% | 27% | 22% | 23% | 22% | 27% |
