# Supplementary material for: Multidrug Resistant Pulmonary Tuberculosis Treatment Regimens and Patient Outcomes: An Individual Patient Data Meta-analysis of 9,153 Patients
Source: PLoS Med. 2012 Aug 28;9(8):e1001300. doi: 10.1371/journal.pmed.1001300 (PMC3429397; doi:10.1371/journal.pmed.1001300)
Supplement: Table S9 — Summary of variance of estimates—individual drugs with treatment success. (DOC) [file pmed.1001300.s017.doc]

**Supplement Table S9:** **Summary of variance of estimates - Individual drugs with Treatment Success**

|  | **Success vs**  **failure/relapse** | | | **Success vs**  **failure/relapse /death** | | | **Success vs failure/relapse /death /default** | | |
| --- | --- | --- | --- | --- | --- | --- | --- | --- | --- |
|  | **N*** | **Intercept**^$^  **Var (SD)** | **Slope**^$^  **Var (SD)** | **N*** | **Intercept**^$^  **Var (SD)** | **Slope**^$^  **Var (SD)** | **N*** | **Intercept**^$^  **Var (SD)** | **Slope**^$^  **Var (SD)** |
| Group 1 Drugs |  |  |  |  |  |  |  |  |  |
| Pyrazinamide | 3985 | 1.23 (0.45) | 0.10 (0.13) | 5096 | 0.86 (0.27) | 0 | 6571 | 0.68 (0.21) | 0.08 (0.06) |
| Ethambutol | 2819 | 1.21 (0.42) | -- | 3740 | 0.76 (0.24) | 0 | 4719 | 0.68 (0.21) | 0.11 (0.06) |
| Group 2: Injectables |  |  |  |  |  |  |  |  |  |
| Kanamycin only | 2860 |  |  | 3437 |  |  | 4457 |  |  |
| vs no injectable |  | 1.60 (0.73) | 0.97 (0.73) |  | 1.51 (0.53) | 1.09 (0.53) |  | 1.36 (0.43) | 1.30 (0.55) |
| vs Capreomycin |  | 1.06 (0.63) | 0.44 (0.54) |  | 0.66 (0.30) | 0.07 (0.13) |  | 0.73 (0.29) | -- |
| vs Streptomycin |  | 0.77 (0.43) | 0.84 (0.61) |  | 0.33 (0.18) | 0.27 (0.21) |  | 0.37 (0.15) | -- |
| Amikacin only | 192 |  |  | 248 |  |  | 307 |  |  |
| vs no injectable |  | 1.45 (0.63) | -- |  | 1.42 (0.51) | 0 |  | 1.52 (0.52) | 1.94 (1.33) |
| Capreomycin only | 769 |  |  | 940 |  |  | 1127 |  |  |
| vs no injectable |  | 1.50 (0.67) | 0.41 (0.61) |  | 1.69 (0.63) | 1.85 (1.41) |  | 1.65 (0.57) | 2.31 (1.22) |
| Group 3: Quinolones |  |  |  |  |  |  |  |  |  |
| Later gen. Quinolones | 751 |  |  | 829 |  |  | 974 |  |  |
| vs no Quinolones |  | 1.83 (0.81) | 1.66 (1.75) |  | 1.53 (0.57) | 0.21 (0.68) |  | 1.33 (0.46) | 0.27 (0.59) |
| vs Ofloxacin |  | 0.25 (0.14) | 1.74 (1.31) |  | 0.59 (0.21) | 0.17 (0.46) |  | 0.75 (0.25) | 0.25 (0.32) |
| vs Ciprofloxacin |  | 1.49 (1.07) | 1.33 (1.89) |  | -- | 1.27 (0.86) |  | 0.16 (0.22) | 1.05 (0.78) |
| Ofloxacin | 3832 |  |  | 4577 |  |  | 6102 |  |  |
| vs no Quinolones |  | 0.93 (0.37) | 0 |  | 1.14 (0.39) | 0.45 (0.28) |  | 1.15 (0.37) | 0.85 (0.37) |
| vs Ciprofloxacin |  | 0.56 (0.26) | 0 |  | 0.47 (0.21) | 0.29 (0.23) |  | 0.40 (0.21) | 0.65 (0.33) |
| Ciprofloxacin | 335 |  |  | 553 |  |  | 644 |  |  |
| vs no Quinolones |  | 2.33 (0.98) | -- |  | 1.45 (0.57) | 0.63 (0.68) |  | 1.25 (0.45) | 1.09 (0.77) |
| Group 4 Drugs |  |  |  |  |  |  |  |  |  |
| Ethionamide/Prothionamide | 4608 | 1.15 (0.41) | 0.10 (0.14) | 5594 | 0.83 (0.26) | -- | 7329 | 0.77 (0.22) | -- |
| Cycloserine/Terizidone | 3547 | 1.22 (0.43) | 0.18 (0.18) | 4194 | 0.95 (0.31) | 0.48 (0.27) | 5358 | 0.85 (0.26) | 0.71 (0.29) |
| Para-aminosalicylic acid (PAS) | 2459 | 1.23 (0.42) | -- | 2860 | 0.78 (0.25) | -- | 3712 | 0.70 (0.21) | 0.09 (0.08) |
| Group 5 Drugs |  |  |  |  |  |  |  |  |  |
| Any 1 Group 5 vs none | 1538 | 1.44 (0.52) | 0.32 (0.27) | 1725 | 0.90 (0.28) | 0 | 2029 | 0.81 (0.24) | -- |
| 2+ Group 5 vs 1 Group 5 | 447 | 1.37 (0.56) | 0.00 (0.17) | 574 | 0.74 (0.32) | 0.00 (0.26) | 654 | 0.41 (0.17) | -- |
| Amox.-Clavulanate only** | 232 | 1.60 (0.78) | 0.14 (0.64) | 255 | 1.07 (0.51) | 0.18 (0.35) | 290 | 0.70 (0.31) | 0.11 (0.28) |
| Clofazimine only** | 651 | 1.26 (0.66) | 1.11 (1.52) | 764 | 0.54 (0.30) | 2.12 (2.01) | 896 | 0.64 (0.35) | 0.26 (0.40) |
| Macrolide only** | 333 | 0.72 (0.29) | -- | 396 | 0.63 (0.22) | 0.02 (0.07) | 459 | 0.58 (0.19) | 0.08 (0.09) |
| Thiacetazone only** | 554 | 0.69 (0.29) | -- | 576 | 0.65 (0.24) | -- | 668 | 0.64 (0.21) | -- |

# aOR; adjusted odds ratios - for use of drug, with non-use as the reference category. Adjusted for age, sex, HIV, past TB treatment, past MDR treatment (treatment for more than 1 month with 2 or more second line drugs), and extent of disease. CI = confidence interval. Missing information was imputed for the following parameters in the following number of patients: Sex was missing in 3, age was missing in 27, HIV was missing in 1271(14%), history of past TB treatment missing in 443 (5%), history of past second line drug use 758 (8%) and extent of disease information missing in 174 (2%).

* N shown is the number of patients that received the drug in question and were included in the specific analysis.

** Group 5 individual drugs: Analysis restricted to patients who received only one Group 5 drug. Each single drug comparison made between patients who received only that Group 5 agent with patients who received any other single Group 5 drug. Drugs included in this analysis as Group 5 drugs were: amoxicillin-clavulanate, macrolides (azithromycin, roxithromycin, and clarithromycin), clofazimine, thiacetazone, imipenem, linezolid, high dose INH, and thioridazine.

Later generation quinolones included levofloxacin, moxifloxacin, gatifloxacin, and sparfloxacin.

Cycloserine included terizidone – a dimer of D-cycloserine given in some centres

^$^Models were fit using random effects logistic regression estimated via penalized quasi-likelihood with random intercepts and random slopes. We report the estimated variance (**Var**) of the random intercepts and slopes and the standard deviation of these estimates.

^^ Both PQL and QUAD gave similar results with only random intercept 0.8 (0.5-1.4). The model estimated via PQL did not converge with random slopes. Results presented are for quadrature with random intercept and slope.
