# Supplementary material for: Multidrug Resistant Pulmonary Tuberculosis Treatment Regimens and Patient Outcomes: An Individual Patient Data Meta-analysis of 9,153 Patients
Source: PLoS Med. 2012 Aug 28;9(8):e1001300. doi: 10.1371/journal.pmed.1001300 (PMC3429397; doi:10.1371/journal.pmed.1001300)
Supplement: Table S10 — Summary of variance of estimates—likely effective drugs with treatment success—during different phases of treatment. (DOC) [file pmed.1001300.s018.doc]

**Supplement Table S10: Summary of variance of estimates - likely effective drugs with treatment success - during different phases of treatment:**

10A: Number of likely effective drugs during Initial intensive phase – all patients – 3 analyses

| Number of drugs | All patients - Success vs Fail/relapse | | All patients - Success vs fail/relapse/death | | All patients - Success vs fail/relapse/death/default | |
| --- | --- | --- | --- | --- | --- | --- |
| . | N | Var (SD) | N | Var (SD) | N | Var (SD) |
| Intercept |  | 1.09 (0.40) |  | 0.82 (0.26) |  | 0.74 (0.22) |
| 0 - 2 | 118 | (reference) | 277 | (reference) | 322 | (reference) |
| 3 | 161 | 0.25 (0.43) | 250 | 0 (--) | 316 | 0.07 (0.11) |
| 4 | 468 | 0.03 (0.25) | 542 | 0 (--) | 671 | 0.13 (0.12) |
| 5 | 814 | 0.14 (0.24) | 900 | 0 (--) | 1114 | 0.25 (0.19) |
| 6+ | 811 | 0.53 (0.45) | 977 | 0 (--) | 1185 | 0 (--) |

10B: Number of likely effective drugs in continuation phase – all patients – 3 analyses

|  | All patients: Success vs  Fail/relapse | | All patients: Success vs  fail/relapse/death | | All patients: Success vs fail/relapse/death/default | |
| --- | --- | --- | --- | --- | --- | --- |
|  | N | Var (SD) | N | Var (SD) | N | Var (SD) |
| Intercept |  | 1.05 (0.38) |  | 1.30 (0.39) |  | 0.98 (0.28) |
| 0 - 2 | 254 | (reference) | 531 | (reference) | 633 | (reference) |
| 3 | 552 | -- | 635 | 0.35 (0.31) | 759 | 0.66 (0.41) |
| 4 | 598 | 0.25 (0.30) | 663 | 0.59 (0.41) | 779 | 0.42 (0.25) |
| 5+ | 560 | 0.53 (0.51) | 608 | -- | 656 | 0.69 (0.44) |

Likely effective: drugs to which isolate susceptible in laboratory testing.

N – Number of patients in subgroup of interest.

aOR; adjusted odds ratios - adjusted for age, sex, HIV, past TB treatment, past MDR treatment (treatment for more than 1 month with two or more second line drugs), and extent of disease. Missing information was imputed for the following parameters in the following number of patients: Sex was missing in 3, age was missing in 27, HIV was missing in 1271(14%), history of past TB treatment missing in 443 (5%), history of past second line drug use 758 (8%) and extent of disease information missing in 174 (2%).

Success: Defined as cure or treatment completion; see Methods for definitions.

Initial intensive phase: period when injectable given. Continuation phase: period when no injectable given.

Only 18 studies provided information regarding drug susceptibility testing and the number of drugs in the initial phase, while only 15 of these described the number of drugs in the continuation phase.

^#^ Random slopes did not converge with PQL. Results were similar to those estimated via QUAD with random intercept and slope though point estimates were slightly lower.

* Similar results to those obtained via QUAD but the last category was not statistically significant.

**With PQL with a random intercept only as random slopes did not converge. Results were very similar to a model fit via QUAD with random intercept and random slopes.

***With PQL with a random intercept only. Random slopes did not converge. Results were similar to those obtained using QUAD with a random intercept.

^##^Calculated by including 4 interaction terms between number of drug (dummy variable) and MDRN status, using a model estimated via PQL with random intercept only as slopes would not converge. Overall p value=0.05. QUAD with random intercept and slope for drugs gave similar results for individual interaction terms but p=0.17 overall.

^$^Using PQL with random intercepts and random slopes. Similar results with Quadrature, though point estimates were a little larger.

^$$^Using PQL with random intercept only. With Quad, random intercept and random slope for 5+ drugs, results were similar, though CIs were much wider.

^$$$^Estimated by including four interaction terms between number of drugs (dummy variable) and MDRN using PQL with random intercepts and slopes. Similar with Quad.
