# Supplementary material for: Multidrug Resistant Pulmonary Tuberculosis Treatment Regimens and Patient Outcomes: An Individual Patient Data Meta-analysis of 9,153 Patients
Source: PLoS Med. 2012 Aug 28;9(8):e1001300. doi: 10.1371/journal.pmed.1001300 (PMC3429397; doi:10.1371/journal.pmed.1001300)
Supplement: Table S11 — Association of number of drugs used and treatment success compared to default only. (DOC) [file pmed.1001300.s019.doc]

**Supplement Tables S11 - Association of number of drugs used and treatment success compared to default only**

**S11A: Number of likely effective drugs in initial phase of treatment**

| Number of drugs | Number with  Success/Default | aOR | (95%CI) |  |
| --- | --- | --- | --- | --- |
| 0 - 2 | 93 / 45 | 1.0 | (reference) |  |
| 3 | 134 / 66 | 1.1 | (0.7, 1.7) |  |
| 4 | 423 / 129 | 1.9 | (1.3, 2.9) |  |
| 5 | 739 / 214 | 1.6 | (1.1, 2.5) |  |
| 6+ | 746 / 208 | 1.4 | (0.9, 2.1) |  |

**S11B: Number of likely effective drugs in continuation phase of treatment**

| Number of drugs | Number with  Success/Default | aOR | (95%CI) |
| --- | --- | --- | --- |
| 0 - 2 | 204 / 50 | 1.0 | (reference) |
| 3 | 513 / 39 | 3.1 | (2.3, 4.1) |
| 4 | 558 / 40 | 4.3 | (2.8, 6.5) |
| 5+ | 510 / 50 | 8.7 | (6.0, 12.6) |

N – Number of patients in subgroup of interest.

aOR; adjusted odds ratios - adjusted for age, sex, HIV, past TB treatment, past MDR treatment (treatment for more than 1 month with two or more second line drugs), and extent of disease

Success: Defined as cure or treatment completion; see Methods for definitions.

Initial intensive phase: period when injectable given. Continuation phase: period when no injectable given.

Only 18 studies provided information regarding drug susceptibility testing and the number of drugs in the initial phase, while only 15 of these described the number of drugs in the continuation phase.
