# Supplementary material for: Multidrug Resistant Pulmonary Tuberculosis Treatment Regimens and Patient Outcomes: An Individual Patient Data Meta-analysis of 9,153 Patients
Source: PLoS Med. 2012 Aug 28;9(8):e1001300. doi: 10.1371/journal.pmed.1001300 (PMC3429397; doi:10.1371/journal.pmed.1001300)
Supplement: Table S12 — Summary of variance of estimates—duration of treatment with success versus failure/relapse—patients grouped by treatment history. (DOC) [file pmed.1001300.s020.doc]

**Supplement Table S12: Summary of variance of estimates - Duration of treatment with success vs Failure/Relapse -**

**Patients grouped by treatment history:**

**S12a:Duration of initial intensive phase.**

| Initial Duration in Months | All patients | | No prior second line drug treatment | | Prior second line drug treatment | |  |
| --- | --- | --- | --- | --- | --- | --- | --- |
|  | N | Var (SD) | N | Var (SD) | N | Var (SD) | P for interaction (Overall=0.11) |
| Intercept |  | 0.90 (0.39) |  | 0.65 (0.43) |  | 0 (--) |  |
| 1 - 2.4 | 308 | -- | 271 | (reference) | 6 | (reference) | -- |
| 2.5 – 3.9 | 1406 | 0.88 (0.78) | 1298 | 0.52 (0.92) | 23 | 0 (--) | 0.81 |
| 4.0 - 5.4 | 481 | 0 (--) | 418 | 0.77 (0.78) | 15 | 0 (--) | 0.94 |
| 5.5 – 6.9 | 377 | 0 (--) | 314 | 0 (--) | 26 | 0 (--) | 0.15 |
| 7.0 - 8.4 | 172 | 0 (--) | 124 | 0 (--) | 21 | 0 (--) | 0.97 |
| 8.5 - 20 | 792 | 0 (--) | 517 | 0.96 (0.98) | 228 | 0 (--) | 0.08 |

**S12b: Total Duration of therapy**

| Total  Duration  In Months | All patients | | No prior second line drug treatment | | Prior second line drug treatment | | P for interaction  (overall=0.10) |
| --- | --- | --- | --- | --- | --- | --- | --- |
|  | N | Var (SD) | N | Var (SD) | N | Var (SD) |  |
| Intercept |  | 1.81 (0.61) |  | 1.57 (0.60) |  | 0.35 (0.26) |  |
| 6.0 - 12.5 | 778 | -- | 681 | (reference) | 33 | (reference) |  |
| 12.6 - 15.5 | 419 | 1.78 (1.06) | 321 | 1.47 (1.00) | 34 | 0 (--) | 0.01 |
| 15.6 - 18.5 | 1700 | 1.81 (1.01) | 1527 | 2.54 (1.51) | 51 | 0 (--) | 0.63 |
| 18.6 - 21.5 | 655 | 2.05 (1.00) | 34 | 2.38 (1.33) | 40 | 0 (--) | 0.09 |
| 21.6 - 24.5 | 553 | 1.47 (0.90) | 400 | 1.16 (0.81) | 105 | 0 (--) | 0.70 |
| 24.6 - 27.5 | 313 | 0.60 (1.06) | 170 | 1.40 (1.87) | 104 | 0 (--) | 0.68 |
| 27.6 – 30.5 | 160 | 1.05 (1.13) | 89 | 0.21 (1.26) | 53 | 0 (--) | 0.27 |
| 30.6 - 36 | 89 | 2.17 (2.02) | 36 | 0.78 (1.41) | 38 | 0 (--) | 0.60 |

N – Number of patients in subgroup of interest.

aOR; adjusted odds ratios - adjusted for age, sex, HIV, past TB treatment, past MDR treatment, and extent of disease. Missing information was imputed for the following parameters in the following number of patients: Sex was missing in 3, age was missing in 27, HIV was missing in 1271(14%), history of past TB treatment missing in 443 (5%), history of past second line drug use 758 (8%) and extent of disease information missing in 174 (2%).

Success: defined as cure or treatment completion and is compared to failure or relapse (see methods for definitions). Other outcomes of death and default not assessed in this analysis because in some data sets shorter duration that was directly due to death or default could not be identified.

Past treatment: Prior MDR means past treatment for more than 1 month with 2 or more second line drugs. No prior MDR includes all other treatment history.

^$^These values are based on plain logistic regression (no random slope, as inclusion of this resulted in a model that did not converge). With quadrature, the variance of the intercept was estimated as 0. ^$$^Estimated via QUAD with a random intercept, as PQl did not converge.
